# Supplementary material for: Dual donor-acceptor covalent organic frameworks for hydrogen peroxide photosynthesis
Source: Nat Commun. 2023 Aug 28;14:5238. doi: 10.1038/s41467-023-40991-7 (PMC10462664; doi:10.1038/s41467-023-40991-7)
Supplement: Supplementary file 1 — Supplementary Information [file 41467_2023_40991_MOESM1_ESM.pdf]

Supplementary Information

Dual donor-acceptor covalent organic frameworks for hydrogen peroxide photosynthesis

Qin et al.

Supplementary Experimental Details

Materials. Benzo[1,2-b:3,4-b':5,6-b'']trithiophene-2,5,8-tricarbaldehyde (Btt), 4,4,4-triaminotriphenylamine (Tapb), 1,3,5-tris(4-aminophenyl)benzene (Tapb), and 2,4,6-tris(4-aminophenyl)-1,3,5-triazine (Tapt) were supplied by Yanshen Technology Co., Ltd. 1,2-Dichlorobenzene (*o*-DCB), 1,4-dioxane, and mesitylene were purchased from Aldric Chemicals. All reagents and solvents were of analytical reagent grade and used as received.

Synthesis of benzo[1,2-b:3,4-b':5,6-b'']trithiophene-2,5,8-tricarbaldehyde (Btt)

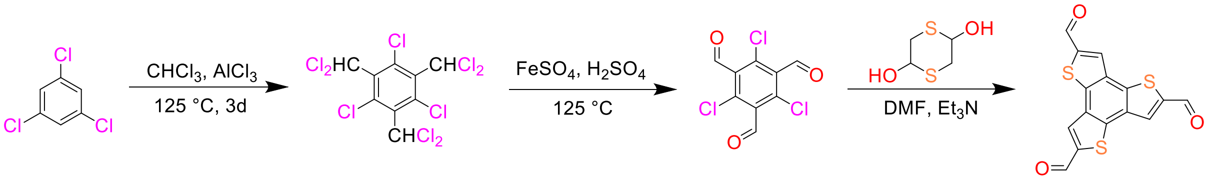

1,3,5-Trichloro-2,4,6-tris(dichloromethyl)benzene was prepared according to a modified literature method[1]. To a 100 mL polytetrafluoroethylene lined reactor was added 1,3,5-trichlorobenzene (3.0 g, 16.5 mmol), aluminium chloride (2.6 g, 19.5 mmol) and trichloromethane (60 mL), and then the mixture was stirred at 125 °C. After 6 h and 18 h of reaction, it was subjected to room temperature and the lining was opened to release the gas. It was cooled down to room temperature and poured into ice water after 3 d. The mixture was stirred for 1 h and extracted with dichloromethane, and solvents were evaporated under reduced pressure. The crude product was chromatographed on silica gel with petroleum ether to get a near white solid (5.56 g, 78.3%) as product. ^{1}H NMR (400 MHz, $CDCl_3$): δ 7.75 (s, 2H), 7.63 (s, 1H) ppm.

1,3,5-Trichloro-2,4,6-tris(dichloromethyl)benzene was prepared according to a modified literature method[1]. To a 25 mL flask was added 1,3,5-trichloro-2,4,6-tris(dichloromethyl)benzene (1.32 g, 3.07 mmol), $FeSO_4$ (60 mg, 0.39 mmol) and concentrated sulfuric acid (8 mL), and the mixture was stirred at 125 °C overnight. After cooling down to room temperature, the reddish-brown oily liquid was poured into ice and stood for a few minutes. Subsequently, the mixture was extracted with CH_2Cl_2 and solvents were evaporated

under reduced pressure. The crude product was chromatographed on silica gel with petroleum ether to get a white solid (0.44 g, 54.0%). ^{1}H NMR (400 MHz, $CDCl_3$): δ 10.42 (s, 3H) ppm.

Benzo[1,2-b:3,4-b':5,6-b'']trithiophene-2,5,8-tricarbaldehyde (BTT) was prepared according to a modified literature method[2]. To a mixture of 2,4,6-trichlorobenzene-1,3,5-tricarbaldehyde (160 mg, 0.6 mmol), pdithiane-2,5-diol (137 mg, 0.9 mmol) and anhydrous DMF (2.2 mL) was added triethylamine (0.5 mL, 3.6 mmol) dropwise in the ice bath. Then, the mixture was stirred at 35 °C overnight. After returning to room temperature, it was poured into ice water and centrifuged, washed with water and THF repeatedly. Finally, a pale brown solid was obtained with 71.6% yield. Due to the poor solubility of the product in common solvents, the structure was indirectly proved through the NMR characterization of its Schiff-based condensation product with 4-tert-butyl benzenamine according to the reported literature[2].

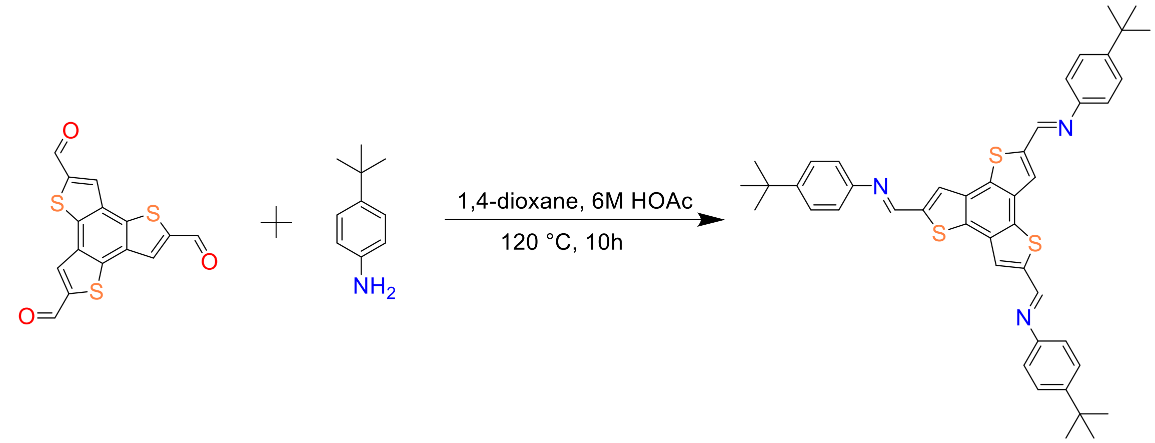

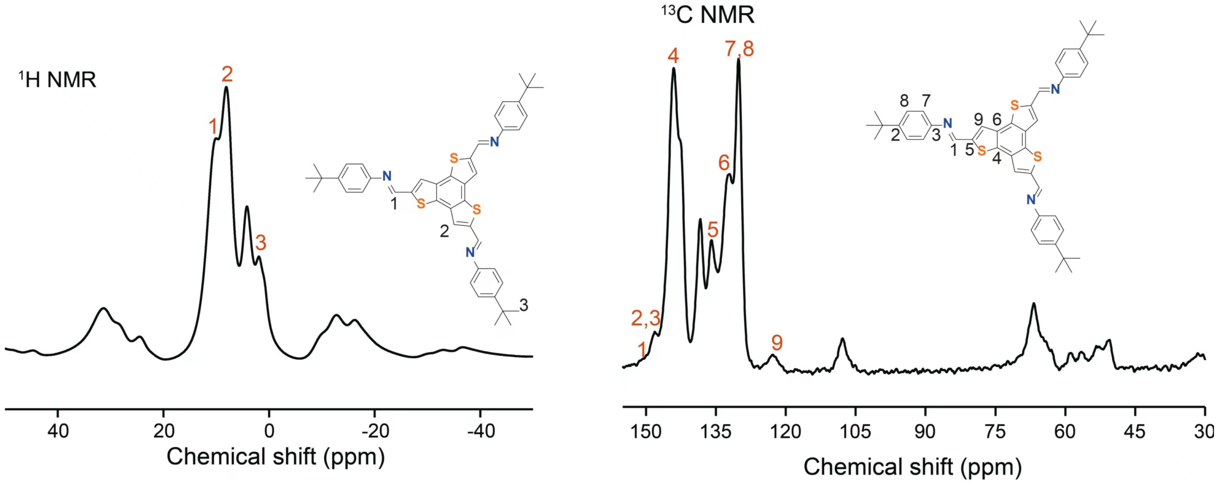

(1E,1'E,1''E)-1,1',1''-(Benzo[1,2-b:3,4-b':5,6-b'']trithiophene-2,5,8-triyl) tris(N-(4(tertbutyl) phenyl)methanimine) (the model compound) was prepared according to a

modified literature method[2]. To a 10 mL flask was added benzo[1,2-b:3,4-b':5,6-b"] trithiophene-2,5,8-tricarbaldehyde (50 mg, 0.15 mmol), 4-tert-butylaniline (86 μL,0.54 mmol), 6M HOAc (0.15 mL) and 1,4-dioxane (4.5 mL), which was degassed three times. The atmosphere was replaced by nitrogen, and the mixture was stirred at 120 °C for 10 h. After cooling to room temperature, the precipitate was filtered and recrystallized in 1,4-dioxane to obtain yellow needle-like solid (66.1 mg, 61%). ^{1}H NMR (400 MHz, $CDCl_3$): δ 9.96 (s, 3H), 8.25 (s, 3H, thiophene), 1.77 (s, 27H, CH_3) ppm. ^{13}C NMR (100 MHz, $CDCl_3$): δ 151.28, 148.02, 143.91, 138.25, 135.70, 131.93, 126.04, 122.52, 35.0, 31.8 ppm.

Preparation of TaptBtt with different crystallinity

TaptBtt-1: 2,4,6-Tris(4-aminophenyl)-1,3,5-triazine (Tapt, 70.8 mg, 0.2 mmol) and benzo[1,2-b:3,4-b':5,6-b"]trithiophene-2,5,8-tricarbaldehyde (Btt, 66.1 mg, 0.2 mmol) were put into 10 mL Pyrex tube, and dissolved into mesitylene (2 mL) and 1,4-dioxane (4 mL) mixed solution (*v*/*v* = 2:4). After the above mixture was sonicated for 10 min, acetic acid aqueous solution (0.5 mL, 6 M) was added, which was then sonicated again for 2 min. The tube was degassed by three freeze-pump-thaw cycles. The tube was sealed off and then heated at 120 °C for 3 days. The powder collected was washed with tetrahydrofuran and acetone for several times, and dried at 80 °C under vacuum for 24 h to obtain TaptBtt-1.

TaptBtt-2: 2,4,6-Tris(4-aminophenyl)-1,3,5-triazine (Tapt, 70.8 mg, 0.2 mmol) and benzo[1,2-b:3,4-b':5,6-b"]trithiophene-2,5,8-tricarbaldehyde (Btt, 66.1 mg, 0.2 mmol) were put into 10 mL Pyrex tube, and dissolved into mesitylene (4 mL) and 1,4-dioxane (2 mL) mixed solution (*v*/*v* = 4:2). After the above mixture was sonicated for 10 min, acetic acid aqueous solution (0.5 mL, 6 M) was added, and then sonicated again for 2 min. The tube was degassed by three freeze-pump-thaw cycles. The tube was sealed off and then heated at 120 °C for 3 days. The powder collected was washed with tetrahydrofuran and acetone for several times, and dried at 80 °C under vacuum for 24 h to obtain TaptBtt-2.

TaptBtt-3: 2,4,6-Tris(4-aminophenyl)-1,3,5-triazine (Tapt, 70.8 mg, 0.2 mmol) and benzo[1,2-b:3,4-b':5,6-b"]trithiophene-2,5,8-tricarbaldehyde (Btt, 66.1 mg, 0.2 mmol) were put

into 10 mL Pyrex tube, and dissolved into mesitylene (6 mL) and 1,4-dioxane (0 mL) mixed solution (*v*/*v* = 6:0). After the above mixture was sonicated for 10 min, acetic acid aqueous solution (0.5 mL, 6 M) was added, and then sonicated again for 2 min. The tube was degassed by three freeze-pump-thaw cycles. The tube was sealed off and then heated at 120 °C for 3 days. The powder collected was washed with tetrahydrofuran and acetone for several times, and dried at 80 °C under vacuum for 24 h to obtain TaptBtt-3.

Synthesis of protonated COFs

Typically, the pristine COFs (50 mg) were stirred in ascorbic acid aqueous solution (50 mL, 0.1 M) for 30 min, followed by natural filtration and drying under vacuum at room temperature overnight.

Synthesis of TpaBtt-fragment (model compound)

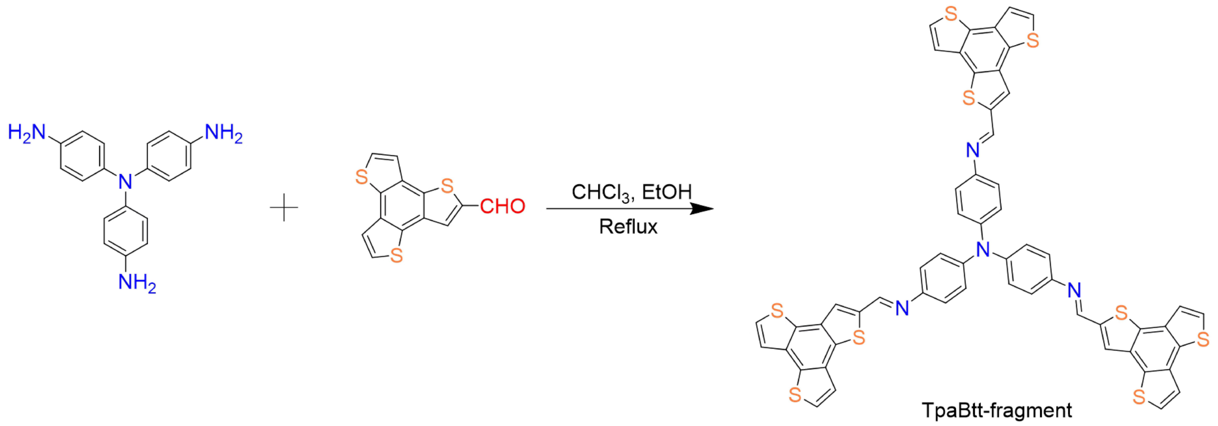

A mixture of 4,4,4-triaminotriphenylamine (0.05 mmol, 14.52 mg) and benzo[1,2-b:3,4-b':5,6-b'']trithiophene-2-tricarbaldehyde (0.2 mmol, 54.88 mg) was dissolved in ethanol (4 mL) and trichloromethane (4 mL), and the mixture was heated to reflux for 24 h. After cooling down to room temperature, the precipitate was collected by filtration, washed with anhydrous ethanol, and dried under vacuum to give a red solid. ^{13}C NMR (δ, *$CDCl_3$*): 152.53, 150.79, 145.31, 142.99, 130.47, 123.56, 65.36.

Synthesis of TapbBtt-fragment (model compound)

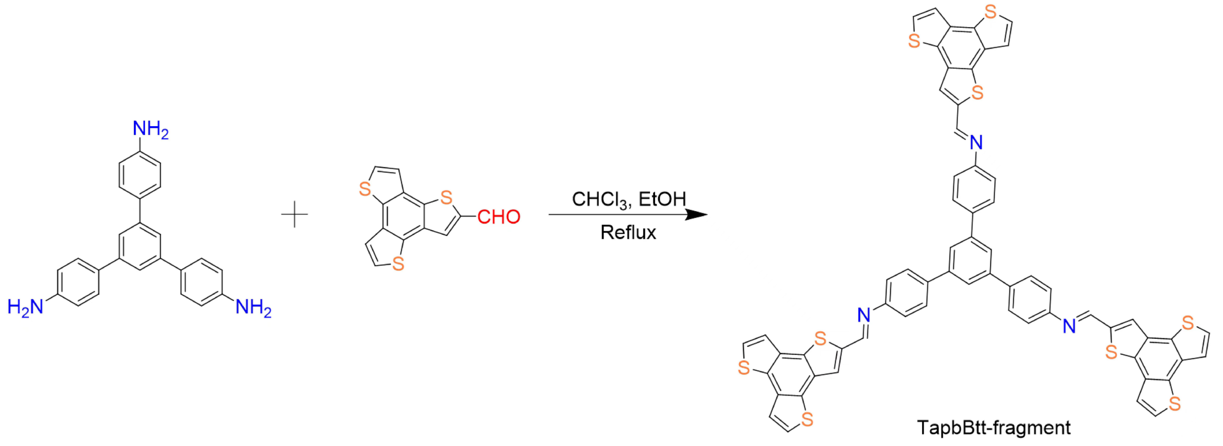

A mixture of 1,3,5-tris(4-aminophenyl) benzene (0.05 mmol, 17.55 mg) and benzo[1,2-b:3,4-b':5,6-b"] trithiophene-2-tricarbaldehyde (0.2 mmol, 54.88 mg) was dissolved in ethanol (4 mL) and trichloromethane (4 mL), and the mixture was heated to reflux for 24 h. After down cooling to room temperature, the precipitate was collected by filtration, washed with anhydrous ethanol, and dried under vacuum to give a red solid. ^{13}C NMR (δ, *$CDCl_3$*): 152.29, 150.44, 148.70, 140.39, 129.31, 125.58, 122.67, 116.01, 112.04.

Synthesis of TaptBtt-fragment (model compound)

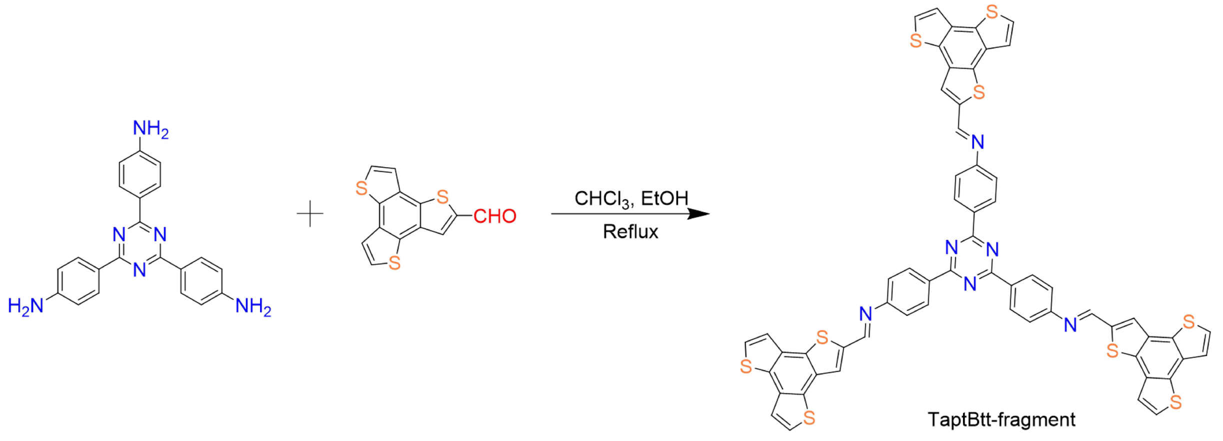

A mixture of 2,4,6-tris(4-aminophenyl)-1,3,5-triazine (0.05 mmol, 17.70 mg) and benzo[1,2-b:3,4-b':5,6-b"]trithiophene-2-tricarbaldehyde (0.2 mmol, 54.88 mg) was dissolved in ethanol (4 mL) and trichloromethane (4 mL), and the mixture was heated to reflux for 24 h. After cooling down to room temperature, the precipitate was collected by filtration, washed with anhydrous ethanol, and dried under vacuum to give a red solid. ^{13}C NMR (δ, *$CDCl_3$*): 169.35, 151.85, 141.93, 132.69, 129.45, 126.06, 122.44, 115.32.

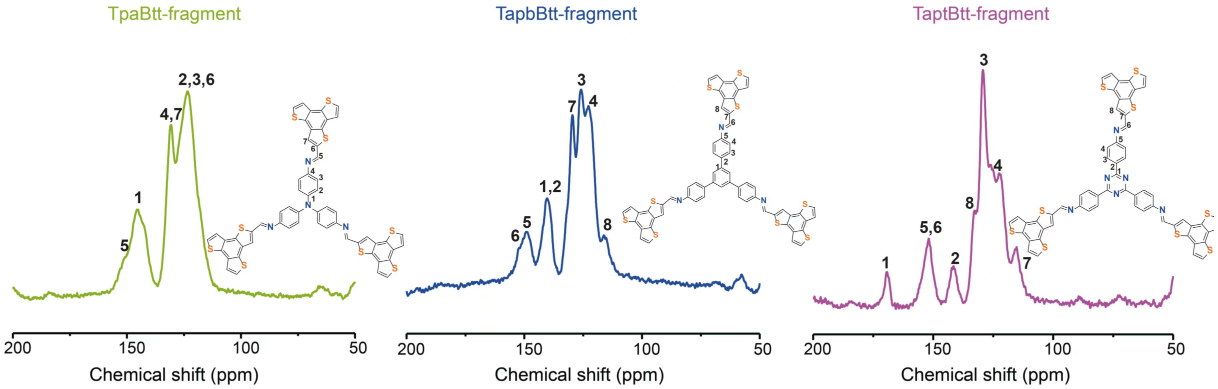

The solid state ^{13}C NMR spectra of three kinds of model compounds showed that the molecular fragments of the three COFs had the feature peaks at 151 ppm, assigning to the carbon of imine bond.

Characterization

Zeiss Sigma 300 XRD patterns were obtained by a Bruker D8 advance powder diffractometer using Ni filtered Cu Kα radiation (3°/min). Scanning electron microscopy (SEM, 300 Sigma, Zeiss) and transmission electron microscopy (TEM, FEL Tecnai G2 F20) were utilized to obtain the morphology of samples. The X-ray photoelectron spectroscopy (XPS) was acquired using a Thermo Scientific K-Alpha spectrometer from Thermo Fisher Scientific. Fourier transform infrared (FT-IR) spectra were recorded on a Thermo Scientific Nicolet iS10 spectrometer between 4000-500 cm^{-1}. The UV-Vis absorption spectra were recorded using PerkinElmer Lambda 750S was used to determine the UV/Visible diffuse reflectance spectrum in the range of 200-800 nm. Photoluminescence (PL) spectroscopy was undertaken using a fluorescence spectrophotometer (Edinburgh, FLS1000). Electron paramagnetic response (EPR) spectra was obtained using Bruker EMXplus-6/1 at room temperature. German Dataphysics OCA20 was used to measure the static contact Angle. The electrochemical impedance and photocurrent response of catalysts were measured on electrochemical workstation (CHI760E, CHI Instruments, Shanghai, China).

Calculation of photoluminescence lifetime

The band-edge emission of three kinds of COF fragments decays by the radiative and

nonradiative electron-hole recombination process. The decay profiles of 350 nm were fitted by a biexponential model[3, 4]:

$$\mathrm{I(t)} = A_1 \exp\left(-t/\tau_1\right) + A_2 \exp\left(-t/\tau_2\right) \qquad (1)$$

$$\tau = \frac{A_1\tau_1^2 + A_2\tau_2^2}{A_1\tau_1 + A_2\tau_2} \qquad (2)$$

where I(t) is intensity, τ, τ_1 and τ_2 are decay times, and A_1 and A_2 can relative magnitudes. By probing the exciton transfer dynamics, the mission decay curves of a material were fitted by biexponential kinetics function, in which two decay components were derived and τ_1 and τ_2 were caused by the nonradiative recombination of charge carrier and the recombination of free excitons, respectively.

H_2O_2 photosynthesis

The experimental procedures of H_2O_2 photosynthesis were similarly carried out according to the literature[5]. Briefly, photocatalyst (15 mg) was added to deionized water (50 mL) in a 100 mL beaker (maximum diameter, φ 60 mm; capacity 100 mL), and the bottle was open at the top. The photocatalyst was dispersed by ultrasonication for 10 min, and then stirred under dark condition for 30 min to reach adsorption-desorption equilibrium at air. The breaker was irradiated at $\lambda > 420$ nm using a 300 W Xe lamp (PLS-SXE300, Beijing Perfectlight) under magnetic and 25 ± 1 °C. After sampling every 15 min, solid-liquid separation procedure was carried out with a filter of 0.22 μm. The residue liquid was detected to the concentration of H_2O_2.

In order to investigate the influence of different sacrificial agents on the H_2O_2 generation activity under the same condition, benzoquinone (BQ, 1 mM), tert-butyl alcohol (TBA, 1%, V/V), CH_3OH (10%) and $KBrO_3$ (1 Mm) were added to the reaction system. To explore the influence of N_2 on the photocatalytic H_2O_2 generation activity, N_2 was continuously injected into the reaction solution under dark conditions for 30 min. After that, the photocatalysis test was carried out under the condition of continuous N_2 flow. A decomposition experiment of H_2O_2 was conducted with catalysts (15 mg) in an aqueous solution (50 mL) containing H_2O_2 (1 mM) under Ar atmosphere. A 300 W Xe lamp (PLS-SXE300, Beijing Perfectlight) was used as the source. The H_2O_2 residue was measured.

H_2O_2 detection

The amount of H_2O_2 was determined by iodometry according to the previous reported method [6]. Specifically, potassium iodide (KI) solution (1 mL, 0.4 mol L^{-1}) and potassium hydrogen phthalate ($C_8H_5KO_4$) solution (1 mL, 0.1 mol L^{-1}) were added to a diluted solution (3 mL) and kept for 30 min. H_2O_2 was reacted with I^- under acidic conditions to form I_3^- ($H_2O_2 + 3I^- + 2H^+ \rightarrow I_3^- + 2H_2O$), which has a strong absorption at about 350 nm by UV-vis spectroscopy. The total amount of H_2O_2 produced during the reaction can be calculated. The following image shows the standard curve of H_2O_2. Calibrating the H_2O_2 concentration by diluting a 30% stock solution was conducted (see below).

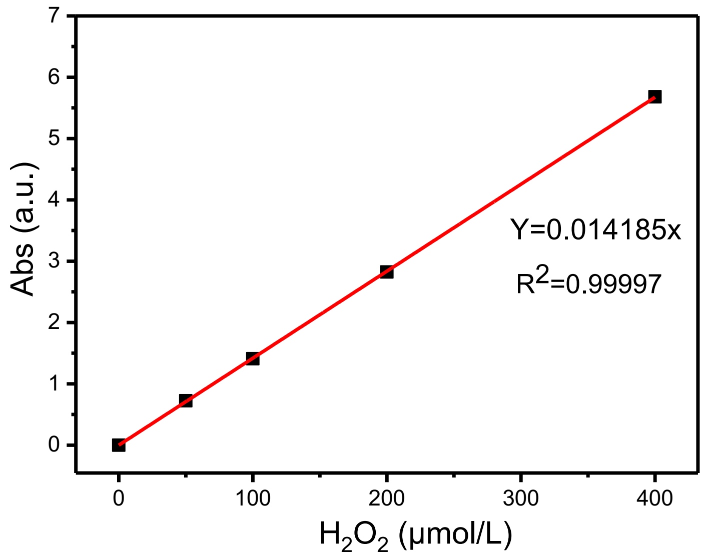

Sulfamethoxazole degradation

Sulfamethoxazole (SMX, 10 mg) and $FeSO_4 \cdot 7H_2O$ (278 mg) were dissolved in a 1 L volumetric flask. Subsequently, photocatalytic H_2O_2 solution (2 mL) was added into SMX stock solution (10 mL). The concentration of SMX at various time periods was measured on Agilent 1260 series high-performance liquid chromatograph (HPLC).

Measurement of apparent quantum efficiency

The measurement of apparent quantum efficiency (AQE) was similarly carried out according to literature [7]. AQE was measured under the illumination of a 300 W Xe lamp (PLS-SXE300, Beijing Perfectlight) with different bandpass of 400, 420, 450, 475 and 520 nm. After ultrasonication and air bubbling, the photocatalytic reaction was carried out in pure deionized

water (60 mL) with photocatalyst (72 mg). The monochromatic light intensity was 12.5 mW cm^{-2}. AQY was calculated by the following formula:

$$AQY = \frac{2 \times H_2O_2\, formed\ (mol)}{the\ number\ of\ incident\ photons\ (mol)} \times 100\% \quad (3)$$

Measurement of solar-to-chemical energy conversion efficiency

According to the experimental method [7], the solar-to-chemical energy conversion (SCC) efficiency was determined by the photocatalytic experiments using an AM 1.5G spectrum as the light source (100 mW cm^{-2}). After air bubbling, the photocatalytic reaction was carried out in pure deionized water (60 mL) with photocatalyst (72 mg). The SCC efficiency was calculated via following equation:

$$SCC(\%) = \frac{[\Delta G\ for\ H_2O_2\ \ generation\ (J\ mol^{-1})][H_2O_2\, formed\ (mol)]}{[total\ input\ power\ (W)][reaction\ times\ (s)]} \times 100\% \quad (4)$$

where $\Delta G = 117$ kJ mol^{-1}. When using TaptBtt as the catalyst, the irradiated sample area was 3.14 cm^2 during the illumination time of 1 h. The calculated total energy (J) was 1130.4 J during the 60 min photocatalytic reaction. Once the x μmol H_2O_2 was generated, the energy generation by H_2O_2 formed was 0.117x J. Therefore, the SCC efficiency can be calculated by the equation of 0.117x/1130.4.

Photoelectrochemical measurements

The photoelectrochemical measurements were conducted by following the literature method [5]. The electrochemical impedance spectroscopy (EIS), photocurrent response, and Mott–Schottky plots were measured on a conventional three electrode potentiostat setup connected to an electrochemical analyzer (Model 760E, CH Instruments) in a 0.2 M Na_2SO_4 aqueous. The catalyst-coated FTO glass of 1 × 2 cm in size was covered with photocatalyst that was obtained by mixing a photocatalyst (3 mg) in Nafion (3 ml, 1%) for 30 min, followed by drying at room temperature and further drying at 60 °C in a vacuum oven. The area of the photoelectrode was controlled to be 0.29 cm^2.

Rotating disk electrode (RDE) measurements

A glassy carbon rotating disk electrode (PINE Research Instrumentation, USA) served as the substrate for working electrode. The detailed processes were according to the literature methods [8, 9]. A glassy carbon disk electrode RDE (0.248 cm^2) was cleaned and exfoliated

samples (50 mg) were dispersed in EtOH (2 mL) containing Nafion (50 μL) by ultrasonication. The slurry (20 μL) was put onto the disk electrode and dried at room temperature. The linear sweep voltammograms (LSV) were obtained in an O_2-saturated 0.1 M phosphate buffer solution (pH = 7) at room temperature with a scan rate 10 mV s^{-1} and different rotation speeds after O_2 bubbling for one hour. The average number of electrons (n) was calculated by Koutecky-Levich equation:

$$\frac{1}{J}=\frac{1}{J_L}+\frac{I}{J_K}=\frac{1}{B\omega^{1/2}}+\frac{1}{J_K} \quad (5)$$

$$B = 0.62nFC_0D_0^{2/3}/3v^{-1/6} \quad (6)$$

where J is the measured current density, J_K and J_L are the kinetic and diffusion-limiting current density respectively, ω is the angular velocity (rad/s), n is transferred electron number, F is Faraday constant (96485 C mol^{-1}), C_0 is the bulk concentration of O_2 (1.26 $\times10^{-6}$ mol cm^{-3}), D_0 is the diffusion coefficient of O_2 in 0.1 M phosphate buffer solution (2.7 $\times10^{-5}$ cm^2s^{-1}), and v is kinetic viscosity of the electrolyte (0.01 cm^2 s^{-1}), respectively.

Rotating ring-disk electrode (RRDE) measurements

A ring-disk electrode (PINE Research Instrumentation, USA) served as the substrate for working electrode. The detailed processes were according to the literature methods [10, 11]. A RRDE electrode with a glassy carbon electrode (0.248 cm^2) and a platinum ring electrode was used as the working electrode (0.187 cm^2). The working electrode was prepared as RDE measurements. The voltammograms were obtained in a 0.1 M phosphate buffer solution (pH = 7) at room temperature under protection of Ar atmosphere with a scan rate of 10 mV s^{-1} and a rotation rate of 1000 rpm. The phosphate buffer electrolyte was purged with Ar for one hour prior to the measurements. The potential of ring electrode was set to -0.23 and 0.6 V (vs. Ag/AgCl) to detect O_2 or H_2O_2, respectively.

Electron paramagnetic resonance (EPR) measurements

Spin trapping EPR tests were recorded using a Bruker EMX plus -6/1 spectrometer operating at the X-band frequency (9.4 GHz). 5,5-Dimethyl-1-pyrroline N-oxide (DMPO) was used as a spin-trapping reagent to detect ·OH or $\cdot O_2^-$. In particular, the catalysts (2 mg) were dispersed into water or a MeOH/water mixture (9/1 *v/v*, 500 μL) containing DMPO (0.1 mmol)

with a Pyrex glass tube, which was sealed with a rubber septum cap. A Xe lamp ($\lambda > 420$ nm) was used as the light source. The dispersion was purged with Ar or O_2 gas for 5 min before light irradiation.

Isotope labelling experiments

The detailed processes of isotope labelling experiments were according to literature method [5]. Catalysts (10 mg) and $H_2^{18}O$ (97%, 1 mL) were put into hermetic device mainly composed of quartz tube and sealing components (the air was pumped away with a vacuum pump). After O_2 was bubbled into the suspension in the dark for 30 min, the suspension was stirred in dark for 30 min to reach the absorption-desorption equilibrium. Prior to the photocatalytic experiment, the O_2 atmosphere was detected by GC-MS as a control. After 6 h irradiation, the gas products in the headspace of the reaction vessel were analyzed by Gas Chromatography-Mass Spectrometer (GC-MS-QP2020 NX, SHIMADZU). Meanwhile, the formed H_2O_2 was decomposed by MnO_2 under Ar atmosphere. The O_2 generated by decomposition of photogenerated H_2O_2 was analyzed by GC-MS. Using this method, we can assess the $H_2{}^{18}O_2$ generated by two-electron water oxidization or two-electron reduction of $^{18}O_2$.

Femtosecond time-resolved transient absorption (fs-TA)

The fs-TA measurements were accomplished using a femtosecond regenerative amplified Ti-sapphire laser system in which the amplifier was seeded with the 120 fs laser pulses from an oscillator laser system. It was measured in the combined utilization of the Femtosecond Laser System (Coherent) and the Helios Pump-Probe System (Ultrafast Systems LLC). The 420 nm pump pulses were generated from optical parametric amplifier (TOPAS-800-fs), which was pumped by the 800 nm fundamental beam outputted from Femtosecond Laser System. The white light continuum probe beam was generated by focusing a small portion (~10 μJ) of the regenerative amplifier's fundamental 800 nm laser pulses into a 2 mm sapphire crystal. The pump and probe beams intersected on the sample at a particular angle. In order to achieve high signal-to-noise ratios, the data were collected with the range of 5 to 10 scans in the measurement and the signal amplitudes in the fs-TA measurements were averaged for further analysis.

***In-situ* diffuse reflectance infrared Fourier transform spectroscopy**

In-situ diffuse reflectance infrared Fourier transform spectroscopy (DRIFTS) measurements were performed based on the literature method[12], using a Bruker IFS 66v Fourier-transform spectrometer equipped with a Harrick diffuse reflectance accessory at the Infrared Spectroscopy and Microspectroscopy Endstation (BL01B). Each spectrum was recorded by averaging 256 scans at a spectral resolution of 4 cm^{-1}. The samples were held in a custom-made IR reaction chamber, which was specifically designed to examine highly scattering powder samples in the diffuse reflection mode. The chamber was sealed with two ZnSe windows.

Photocatalytic evolution of $\cdot O_2^-$

The concentration of $\cdot O_2^-$ was measured by detecting the decay of nitro blue tetrazolium (NBT) according to the literature[13]. Liquid (3 mL) was filtrated with a 0.22 μm filter to remove the photocatalysts. The photocatalytic generation of $\cdot O_2^-$ was determined by the degradation of NBT, which was detected by the absorbance change at the wavelength of 259 nm using UV-vis spectroscopy. The mole ratio of generated $\cdot O_2^-$ and reacted NBT was 4:1 according to the following equation.

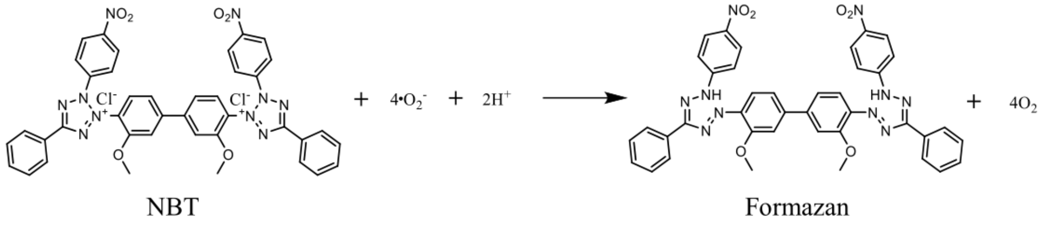

Theoretical computation details

All-electron density functional theory (DFT) calculations were carried out by the ORCA quantum chemistry software (Version 5.0.3). Grimme's D3BJ dispersion correction was used to improve calculation accuracy[14]. The excited states were calculated with linear response time-dependent DFT (TD-DFT) at the optimized ground state geometry. The excited states calculations were using the CAM-B3LYP functional and the def2-SVP basis set[15-18].

Structural optimization and electronic structures were performed using the spin unrestricted Density Functional Theory (DFT) as implemented in Dmol3 package. The

generalized gradient approximation (GGA) of Perdew–Burke–Ernzerhof (PBE) functional was used for describing the exchange-correlation potential and the double-numerical basis set (DNP) was also employed in this work. A 15 Å vacuum space was involved along the *z*-direction to safely ensure decoupling between each slab caused by the periodical condition. During the geometry optimization process, a 4×4×1 Monkhorst-Pack *k*-points mesh was used for sampling the Brillouin zone, and the threshold was set to be 1.0×10^{-5} Hartree, 2.0×10^{-3} Hartree/Å and 5.0×10^{-3} Å for energy, maximum force and maximum displacement, respectively. A 4×4×1 k-points mesh was also used for electronic structures calculations in this work. Periodic DFT calculations were performed to verify the capability of O_2 adsorption and the change of the Gibbs free energy along the path of O_2 to H_2O_2.

The free energy of a gas phase molecule or an adsorbate on the surface was calculated by the equation G = E + ZPE − TS, where E is the total energy, ZPE is the zero-point energy, T is the temperature in kelvin (298.15 K is set here), and S is the entropy. The process of $2e^-$ ORR (Eq. 7-10) or 2 e^- WOR (Eq. 11-12) proceeds based on the following equations:

$$* + O_2\,(g) \rightarrow O_2* \quad (7)$$

$$O_2* + H^+ + e^- \rightarrow O_2* + H* \quad (8)$$

$$O_2* + H^* \rightarrow OOH^* \quad (9)$$

$$OOH^* + H^+ + e^- \rightarrow H_2O_2* \quad (10)$$

$$H_2O + h^+ \rightarrow OH^* + H^+ \quad (11)$$

$$OH^* + OH^* \rightarrow H_2O_2* \quad (12)$$

where * represents the surface-active site.

The photocatalytic reduction of O_2 to H_2O_2 can be divided into four steps. i) O_2 is chemically adsorbed and active to O_2* intermediate. ii) O_2* intermediate is hydrogenated to O_2*/H*. iii) O_2*/H* intermediates form OOH*. iv) OOH* intermediate is continuously hydrogenated to H_2O_2 and desorbs from the catalyst.

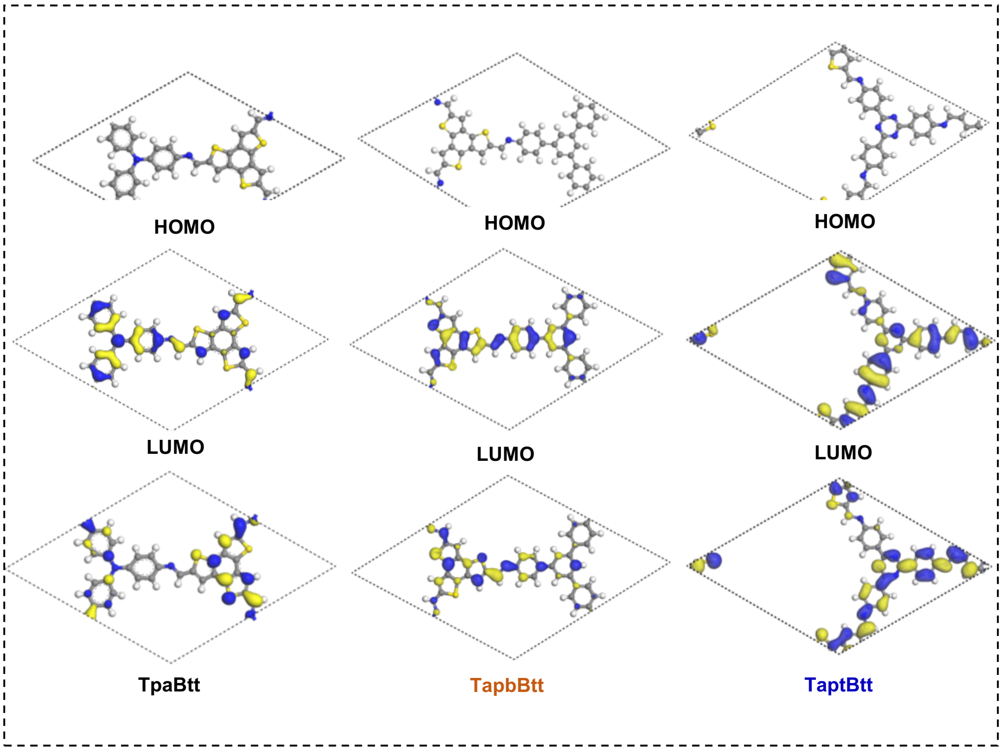

Supplementary Figure 1. Calculated HOMO-LUMO distribution of three COFs.

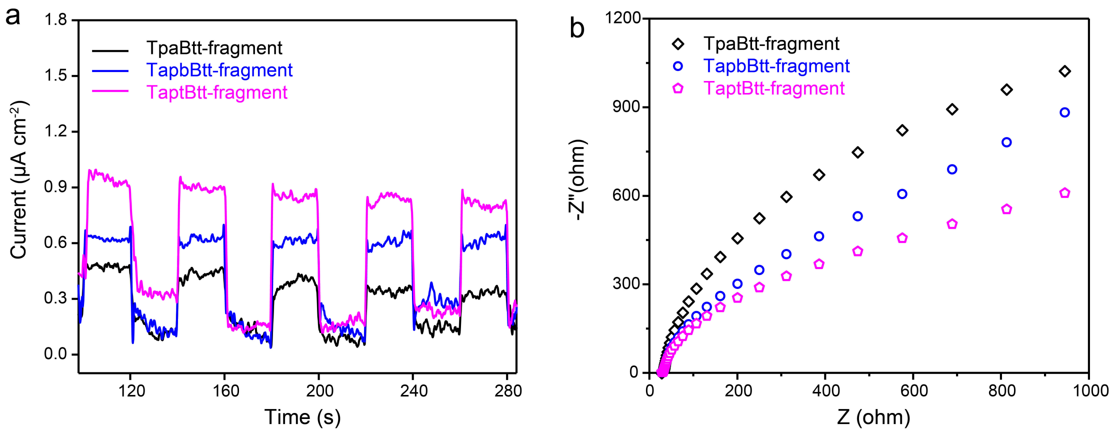

Supplementary Figure 2. **Transient photocurrent response and EIS results of fragment.** (a) Transient photocurrent responses under visible light irradiation, and (b) electrochemical impedance spectroscopy (EIS) of fragments.

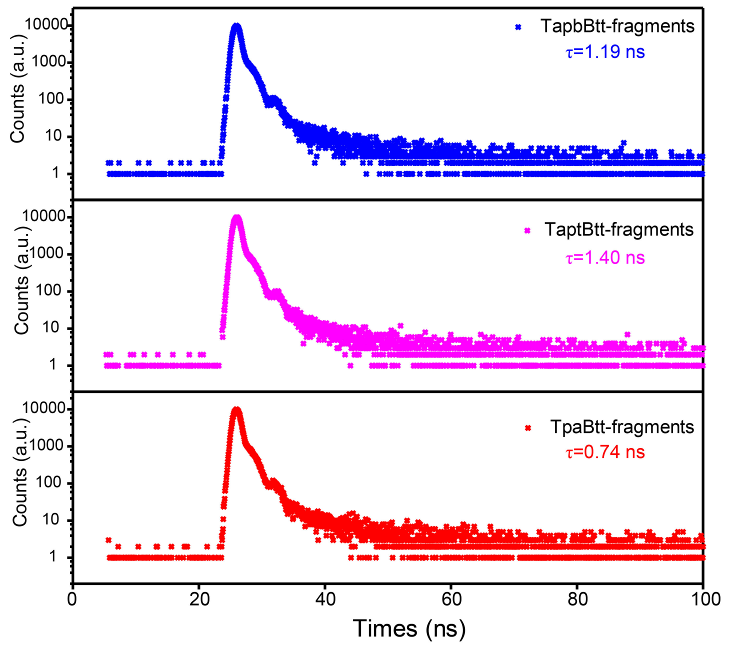

Supplementary Figure 3. Fluorescence lifetime decay of three kinds of COFs fragments. The fluorescence lifetime of TaptBtt-fragment (1.40 ns) excited at 350 nm was obviously higher than that of TapbBtt-fragment (1.19 ns) and TpaBtt-fragment (0.74 ns), indicating that TaptBtt-fragment was able to perform charge separation and suppress e^{-}-h^{+} recombination more effectively than TapbBtt-fragments and TpaBtt-fragments[19].

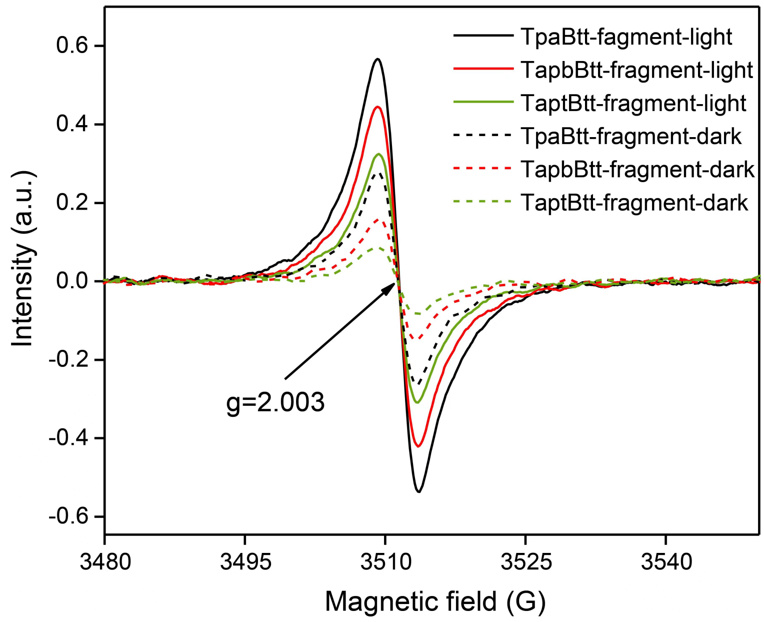

Supplementary Figure 4. EPR spectra of all COF fragments under dark and light.

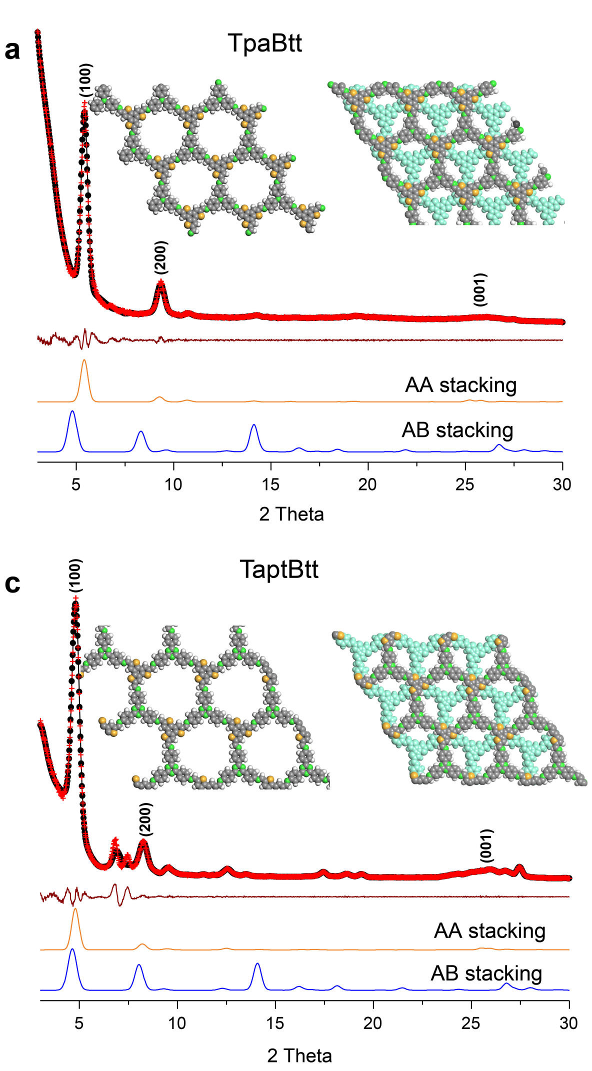

Supplementary Figure 5. PXRD patterns and simulated stacking models. Experimental, refined and simulated PXRD patterns of (a) TpaBtt, (b) TapbBtt and (c) TaptBtt. Inserts represented the simulated eclipsed (AA) and staggered (AB) stacking models of the corresponding imine 2D COFs. Material Studio software was used to simulate the crystal structure, and the Pawley refinements demonstrated the good fit of the eclipse stacking model (AA stacking) for three COFs. The optimized PXRD displayed TpaBtt, TapbBtt and TaptBtt the (100) refection at 2θ = 5.43°, 4.84° and 4.76°. Pawley refined AA stacking model based on the experimental profiles gave unit cells with parameters (a = b = 19.1881 Å, c = 3.5238, α = β = 90°, γ = 120°) for TpaBtt (residuals Rp = 3..01%, Rwp = 4.05%), (a = b = 21.350 Å, c = 3.505, α = β = 90°, γ = 120°) for TapbBtt (residuals Rp = 2.75%, Rwp = 4.55%), and (a = b = 21.755 Å, c = 3.493, α = β = 90°, γ = 120°) for TaptBtt (residuals Rp = 3.99%, Rwp = 8.63%).

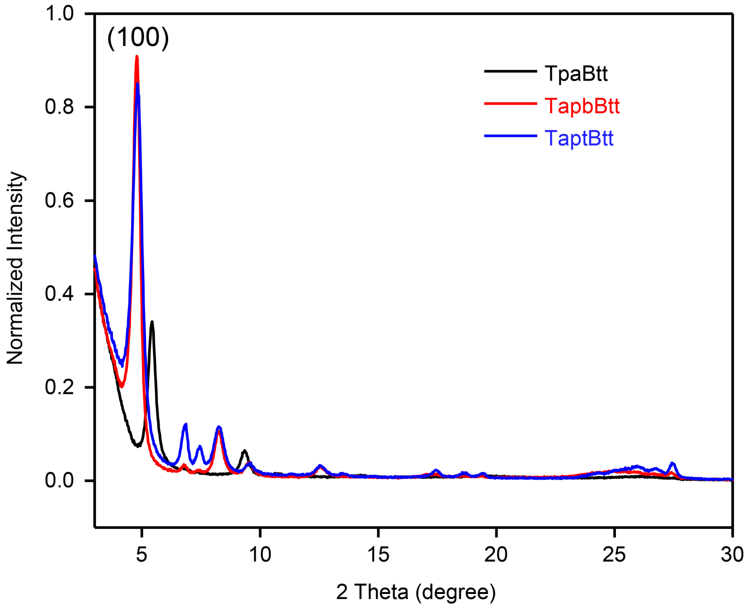

Supplementary Figure 6. Normalized PXRD profiles of TpaBtt, TapbBtt and TaptBtt.

Supplementary Note 1:

The full width at half maximum (FWHM) correlates with the crystal size according to the Scherrer equation[20]:

$$D = \frac{K\lambda}{\beta COS\theta} \quad (13)$$

where D is the mean size of the ordered (crystalline) domains, which may be smaller or equal to the grain size. K is a dimensionless shape factor, with a value close to unity. The shape factor has a typical value of about 0.9, but varies with the actual shape of the crystallite. λ is the X-ray wavelength. β is the line broadening at half the maximum intensity (FWHM) in radian, after subtracting the instrumental line broadening. This quantity is also denoted as Δ (2θ). θ is the Bragg angle.

According to the Scherrer equation, the bigger β value is, the more ordered (crystalline) domains present in the framework. The higher degree of ordered domains of crystal is, the larger range of electron delocalized in the skeleton. Therefore, the FWHM of the principle (100) peaks in the PXRD profiles of three kinds of COFs were compared. After normalizing the PXRD intensity (**Supplementary Figure 6**), the (100) peak of TpaBtt, TapbBtt and TaptBtt has a FWHM of 0.51°, 0.39° and 0.47°, respectively. The microcrystal sizes of TpaBtt, TapbBtt and TaptBtt are 15.45, 20.30 and 16.81 nm, respectively. The TapbBtt exhibited the minimum

FWHM in all of COFs, indicating that it has the maximum π-conjugated degree of ordered domains.

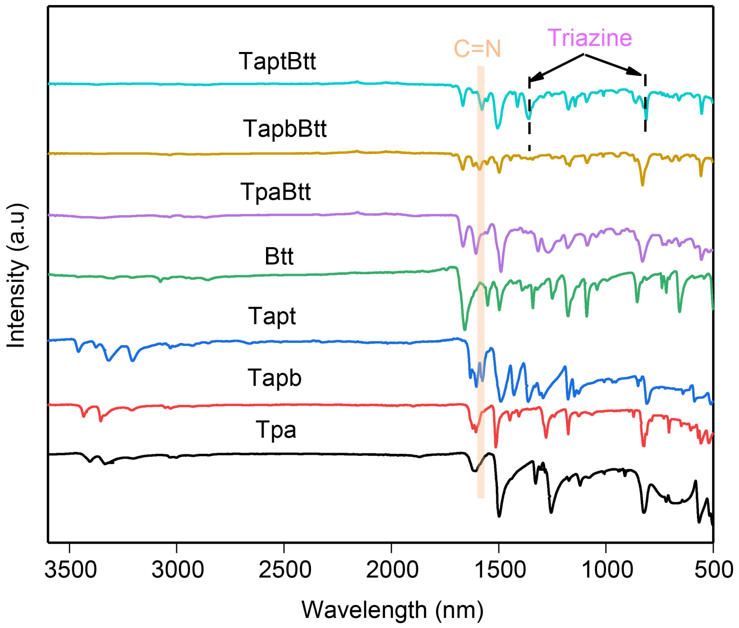

Supplementary Figure 7. FTIR spectra of TpaBtt, TapbBtt and TaptBtt with corresponding organic building blocks.

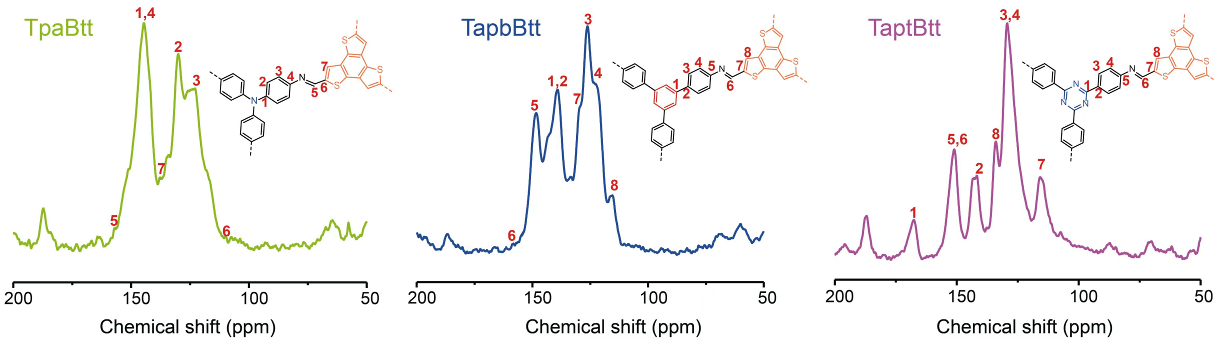

Supplementary Figure 8. Solid state ^{13}C CP/MAS NMR spectra of these COFs.

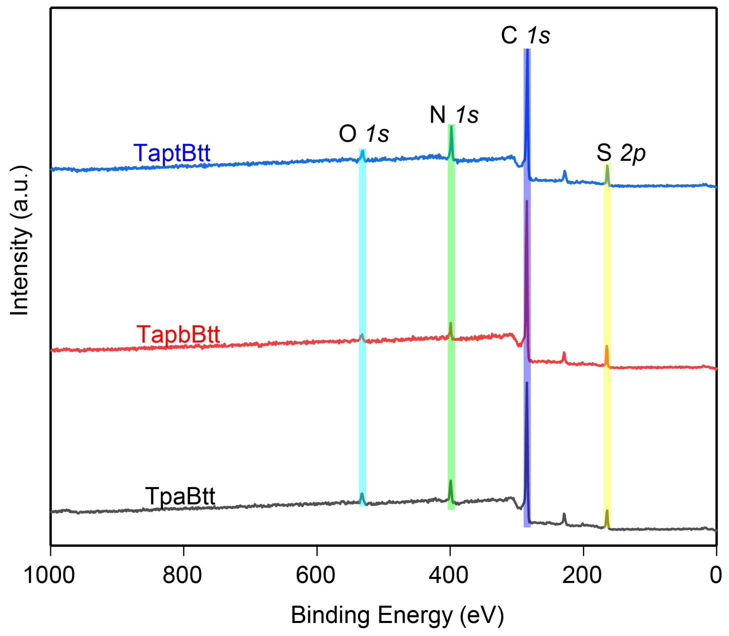

Supplementary Figure 9. XPS survey spectra of TpaBtt, TapbBtt and TaptBtt.

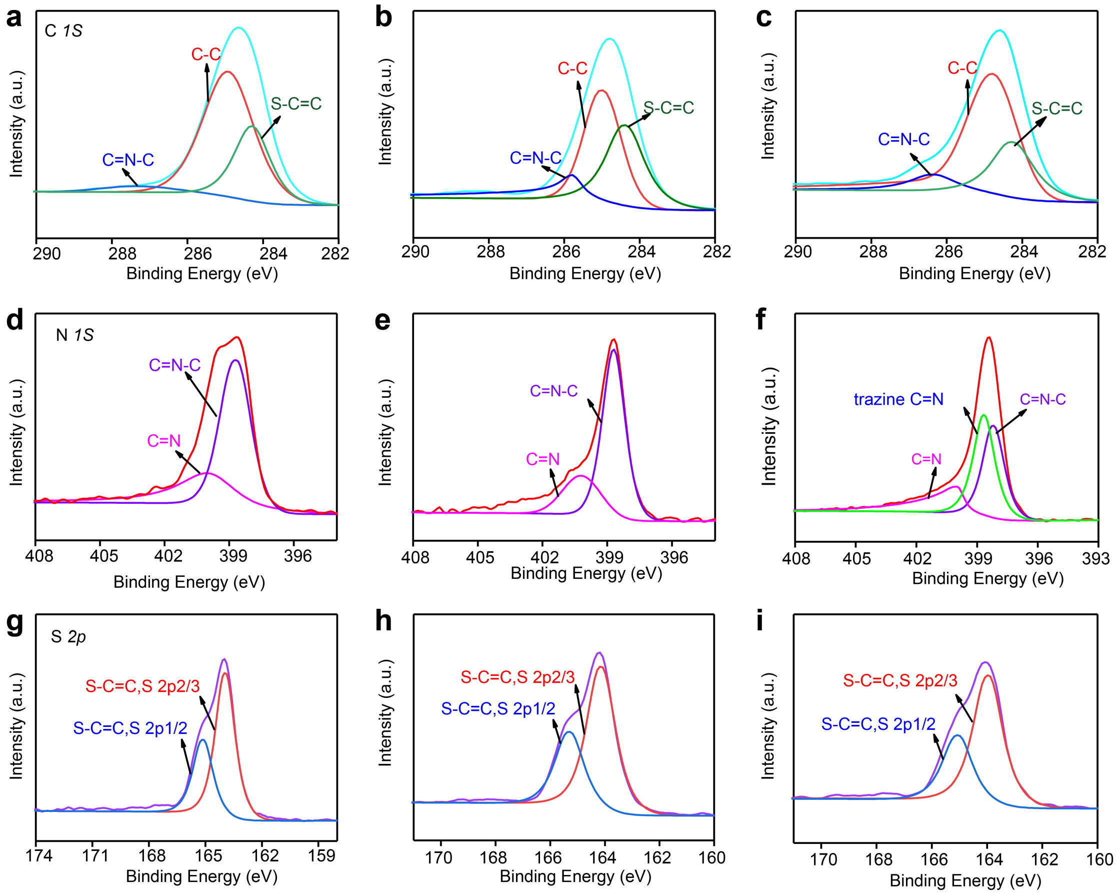

Supplementary Figure 10. XPS spectra. (a-c) C 1s, (d-f) N 1s, and (g-i) S 2p for TpaBtt, TapbBtt and TaptBtt.

Supplementary Note 2:

In order to prove the element types and chemical states on the surface of three COFs, the XPS spectra were obtained (**Supplementary Figure 10**). The characteristic peaks of C1s, N1s and S 2p are attributed to the carbon, nitrogen and sulfur elements in the COFs. The C1s XPS profile of TpaBtt (**Supplementary Figure 10a**), TapbBtt (**Supplementary Figure 10b**) and TaptBtt (**Supplementary Figure 10c**) is deconvoluted into three peaks at binding energy of 284.28, 284.28 and 284.38 eV (sp^2 aromatic carbon), 284.98, 284.98 and 284.78 eV (C=N bond in imine bond), 287.28, 285.78 and 286.38 eV (S-C=C bond belong to the thiophene ring), respectively [21]. The N1s peak is divided two peaks for TpaBtt (**Supplementary Figure 10d**) and TapbBtt (**Supplementary Figure 10e**), located at 398.68 and 400.18 eV, which should be attributed to the C=N (imine bond) and C-N bond in the triphenylamine [22]. For TaptBtt in **Supplementary Figure 10f**, triazine C=N has a clear peak at the 398.68 eV. In the S 2p region, the two sets of peaks S 2p3/2 and S 2p1/2 spin orbitals of sulfur (S-C=C) were observed, with binding energies of 163.98 and 165.08 eV for TpaBtt (**Supplementary Figure 10g**), 164.18 and 165.28 eV for TapbBtt (**Supplementary Figure 10h**), and 163.98 and 165.08 eV (**Supplementary Figure 10i**) for TaptBtt.

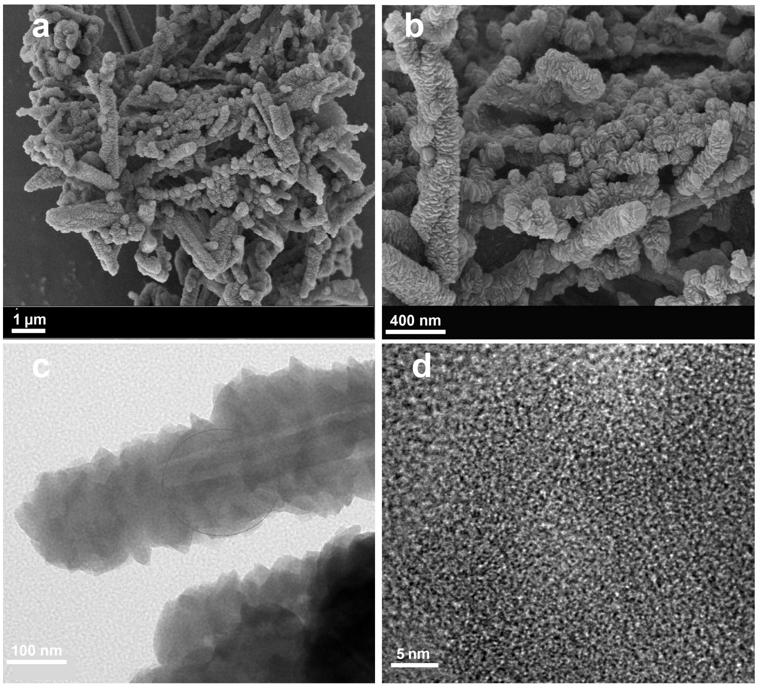

Supplementary Figure 11. SEM and TEM images. (a,b) SEM images and (c,d) TEM images of TpaBtt.

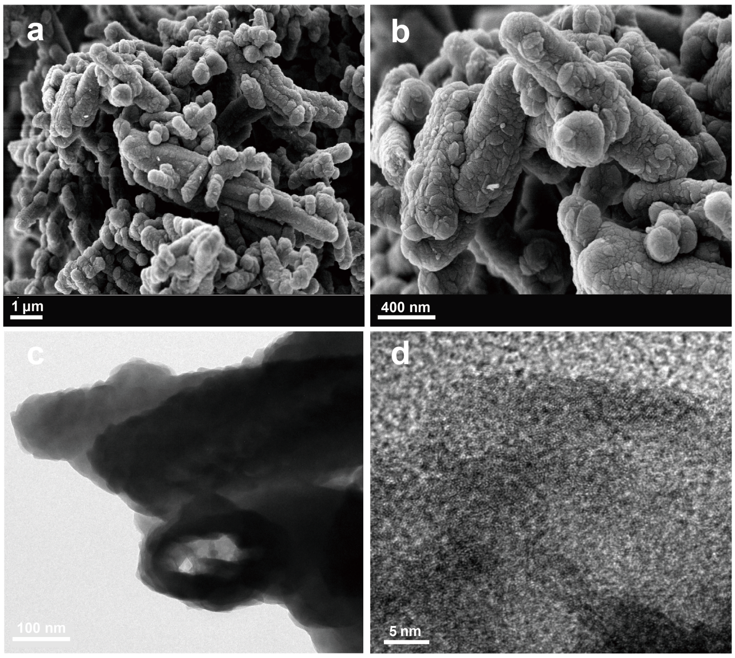

Supplementary Figure 12. SEM and TEM images. (a,b) SEM images and (c,d) TEM images of TapbBtt.

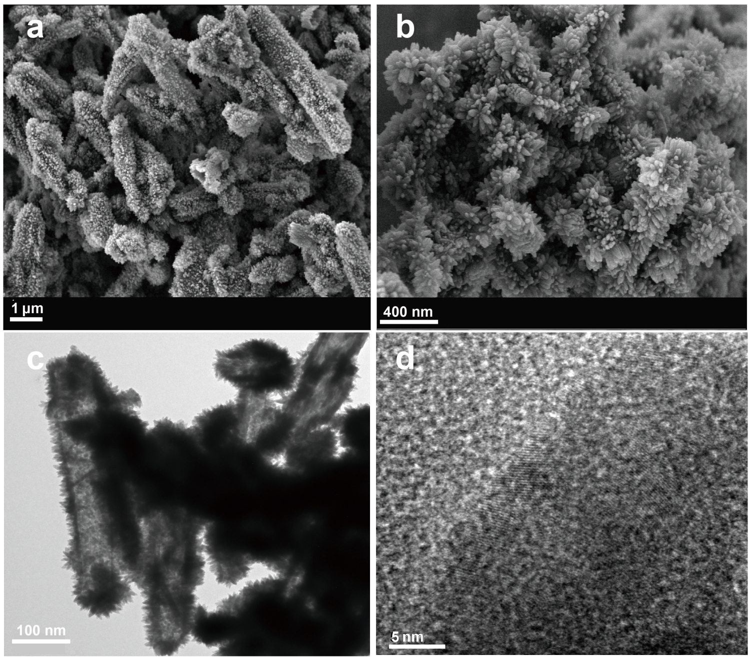

Supplementary Figure 13. SEM and TEM images. (a,b) SEM images and (c,d) TEM images of TaptBtt.

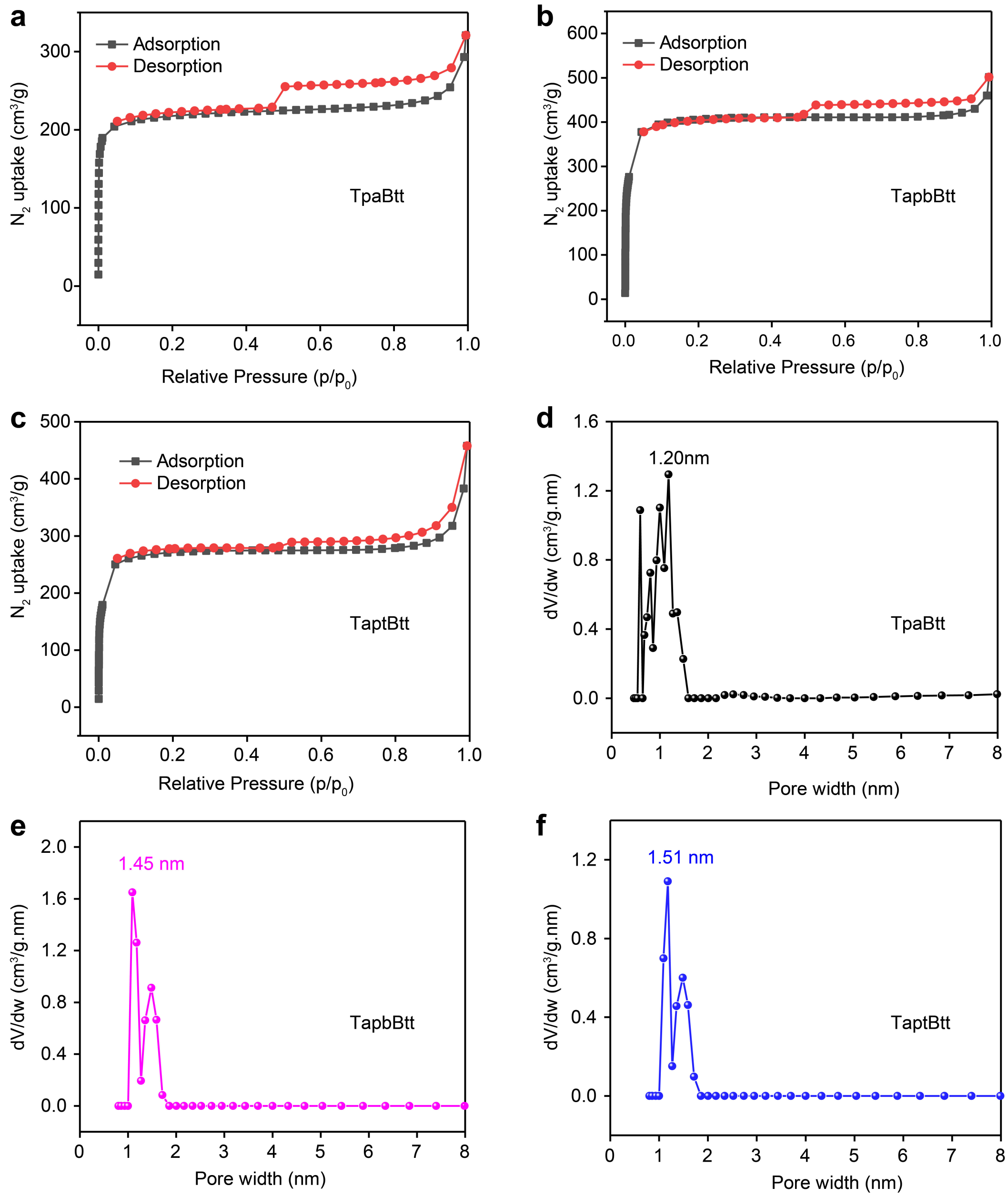

Supplementary Figure 14. N_2 adsorption-desorption isotherms and pore distribution. (a,d) TpaBtt, (b,e) TapbBtt and (c,f) TaptBtt.

Supplementary Note 3:

The SEM images showed that both TpaBtt (**Supplementary Figure 11a,b**) and TapbBtt (**Supplementary Figure 11a,b**) had aggregates with branches, and the tubular branch has a thin wall and a hollow part based on the TEM images (**Supplementary Figure 11c** and **12c**). Both the SEM (**Supplementary Figure 13a,b**) and TEM images (**Supplementary Figure 13c**) for

TaptBtt showed a shape similar to a hollow sea urchin with raised burrs around it, which may accelerate the contact of the active site with oxygen and water, and promote the generation of hydrogen peroxide. TEM images confirm that both TpaBtt (**Supplementary Figure 11d**) and TapbBtt (**Supplementary Figure 12d**) crystals have staggered linear morphology, and there are a large number of aggregated single crystals, especially for TpaBtt. All of images presented regularly and alternately dark and bright dots, indicating the open-porous structure. As shown in the image (**Supplementary Figure 13d**), TaptBtt with high crystallinity can be observed with distinct lattice stripes compared with the other two COFs. The sharp rise in the low-pressure range, as a character of the type I sorption isotherm, indicates the microporous nature (**Supplementary Figure 14).** The pore volume at P/P_0=0.99 increases in the order of TpaBtt (0.42 cm^3 g^{-1}) < TaptBtt (0.53 cm^3 g^{-1}) < TaptBtt (0.71 cm^3 g^{-1}), and micropore volume is 0.28, 0.55 and 0.36 cm^3 g^{-1} for TpaBtt, TapbBtt and TaptBtt, respectively (**Supplementary Table 3**).

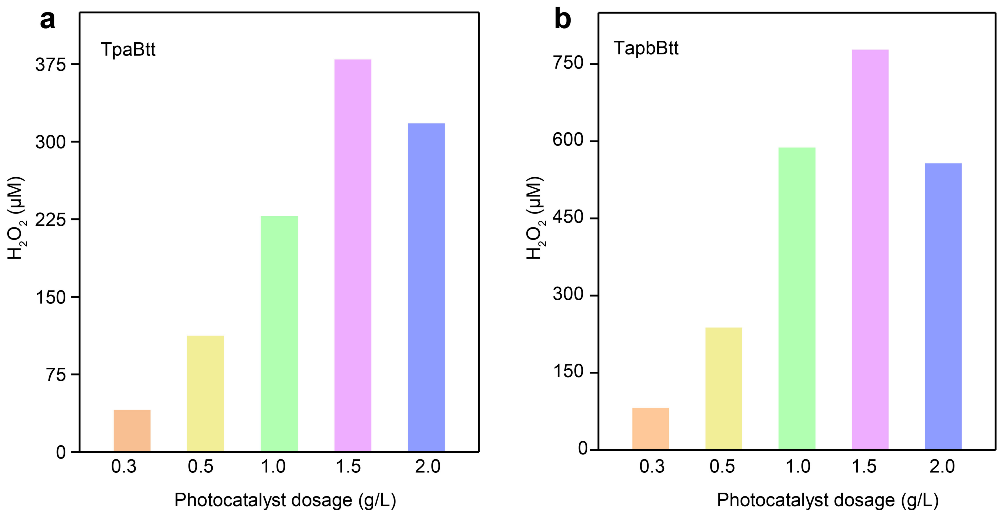

Supplementary Figure 15. Photocatalytic activity. Photocatalytic activity of (a) TpaBtt and (b) TapbBtt with different catalyst concentrations (conditions: 15mg catalyst and 50 mL pure water, 300 W Xe lamp, λ > 420 nm).

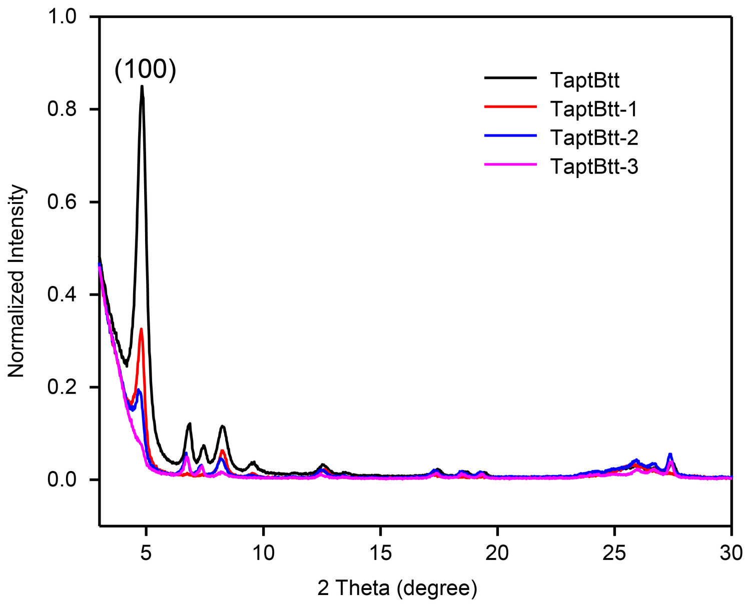

Supplementary Figure 16. Normalized PXRD profiles of TaptBtt, TaptBtt-1, TaptBtt-2 and TaptBtt-3.

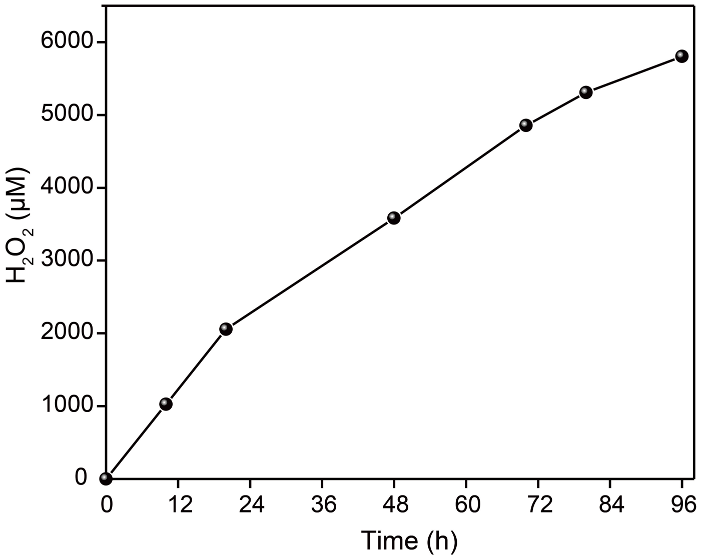

Supplementary Figure 17. Long-term photocatalytic H_2O_2 production of TaptBtt: 100 mL of pure water and 80 mg of TaptBtt, 300W Xe lamp, λ > 420 nm.

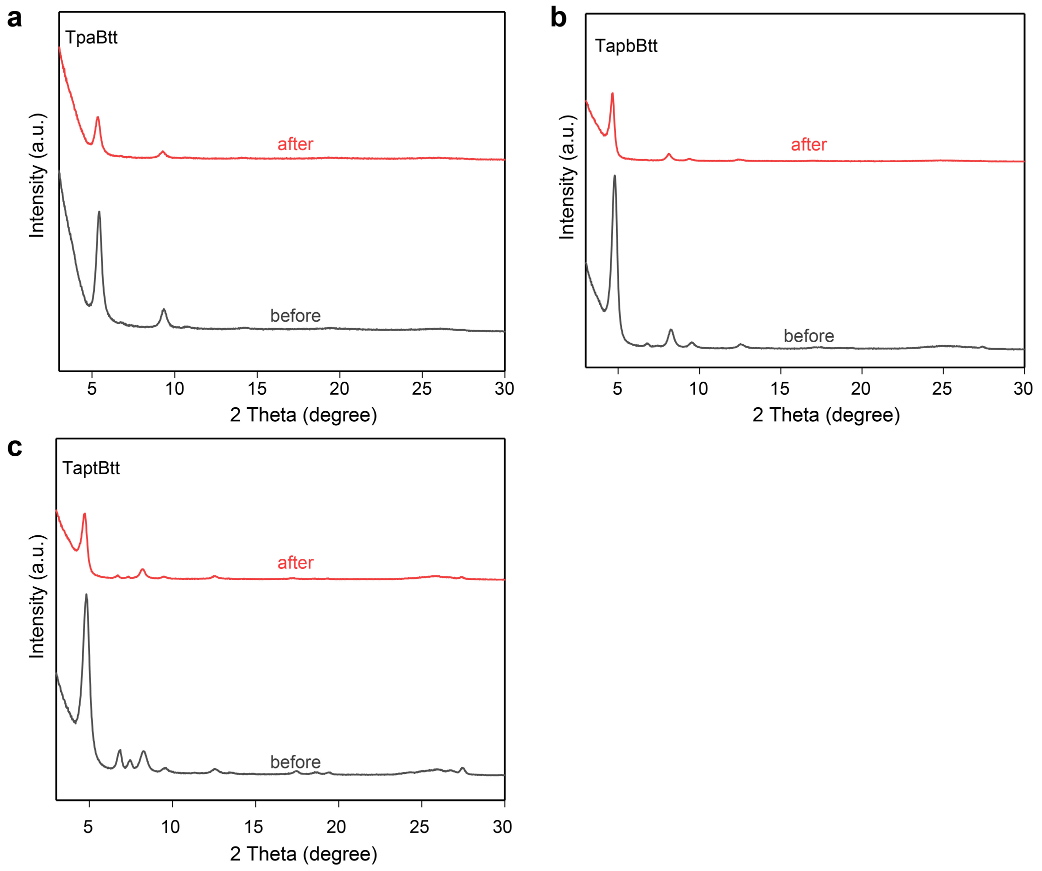

Supplementary Figure 18. PXRD patterns before and after photocatalytic reaction. PXRD patterns of (a) TpaBtt, (b) TapbBtt and (c) TaptBtt before and after photocatalytic reaction. The crystalline structures before and after photocatalytic reactions are still preserved for all of the three COFs.

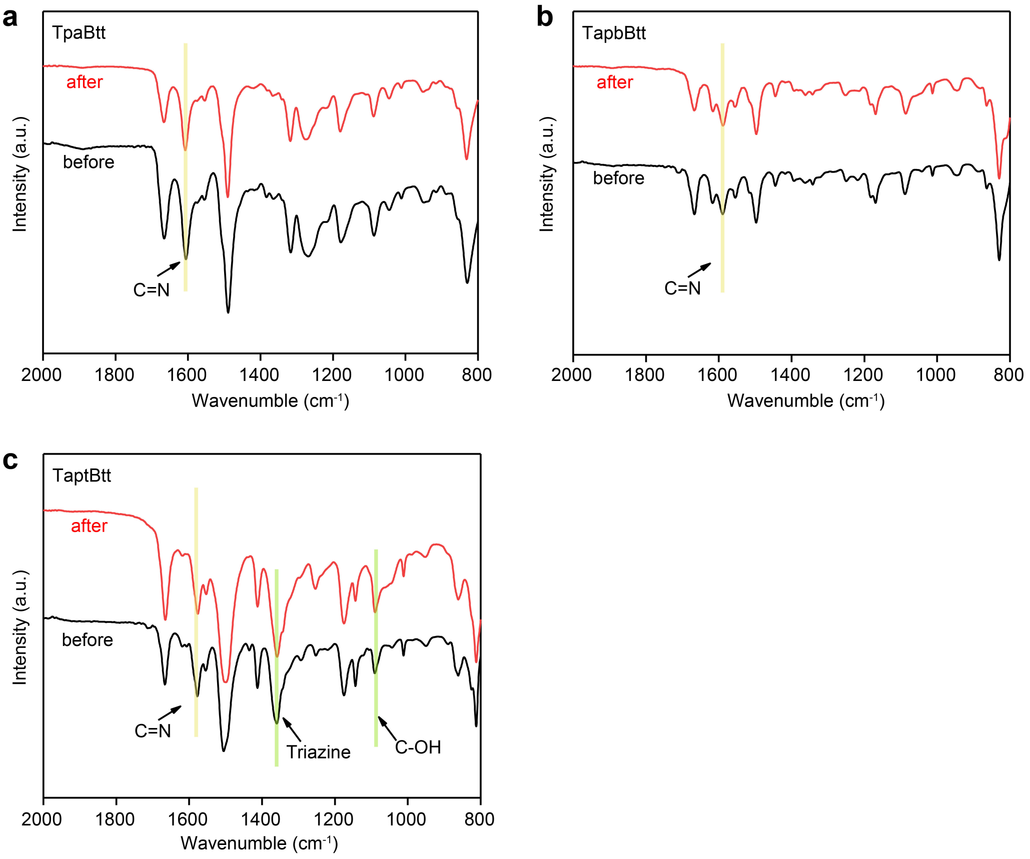

Supplementary Figure 19. FITR spectra before and after photocatalytic reaction. FITR spectra of (a) TpaBtt, (b) TapbBtt and (c) TaptBtt before and after photocatalytic reaction. The imine bond was preserved before and after the reaction for the three COFs, and the strength of triazine unit (1355 cm^{-1}) increased after the reaction for TaptBtt.

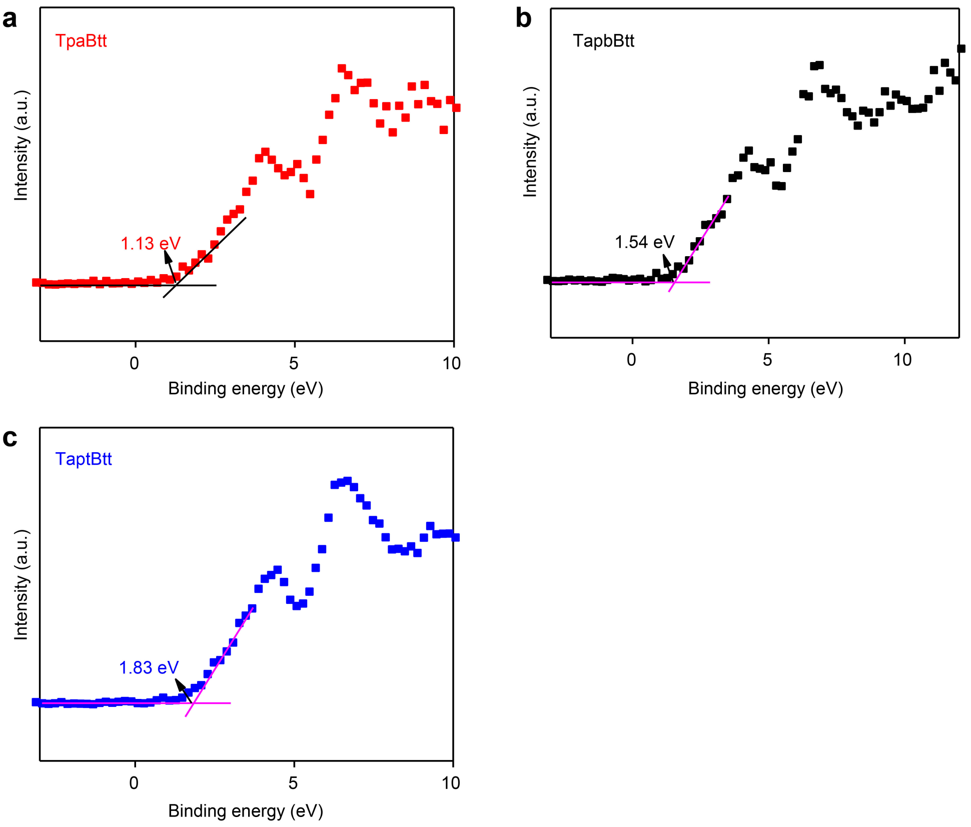

Supplementary Figure 20. VB-XPS spectra. (a) TpaBtt, (b) TapbBtt and (c) TaptBtt.

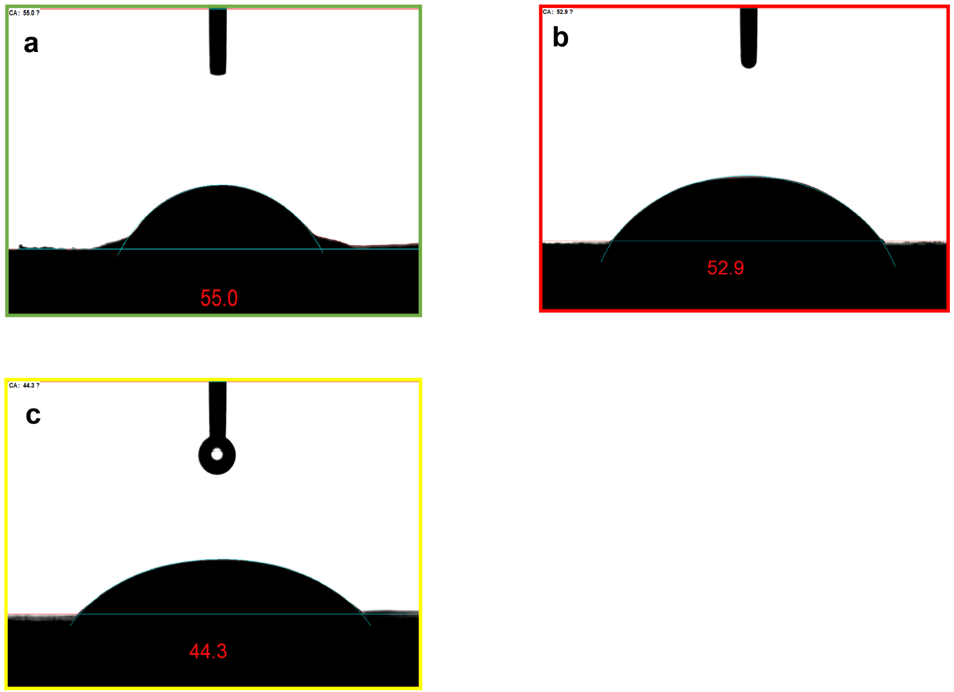

Supplementary Figure 21. Contact angles. (a) TpaBtt, (b) TapbBtt and (c) TaptBtt.

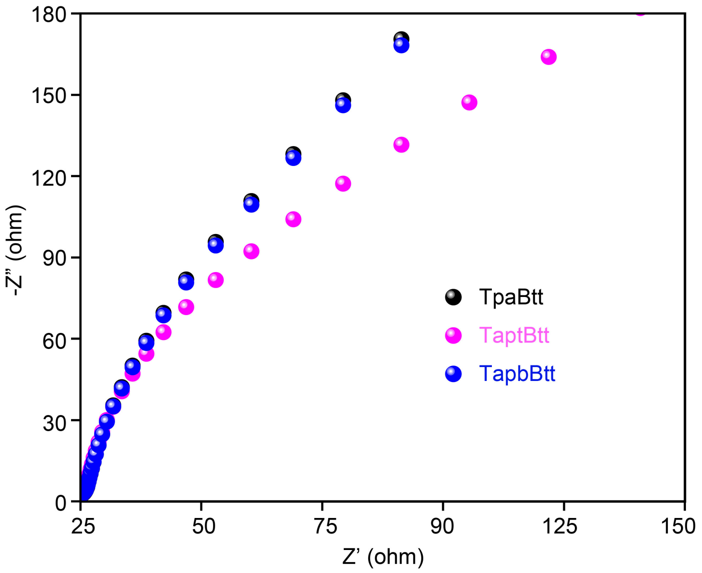

Supplementary Figure 22. Electrochemical impedance spectra of three COFs.

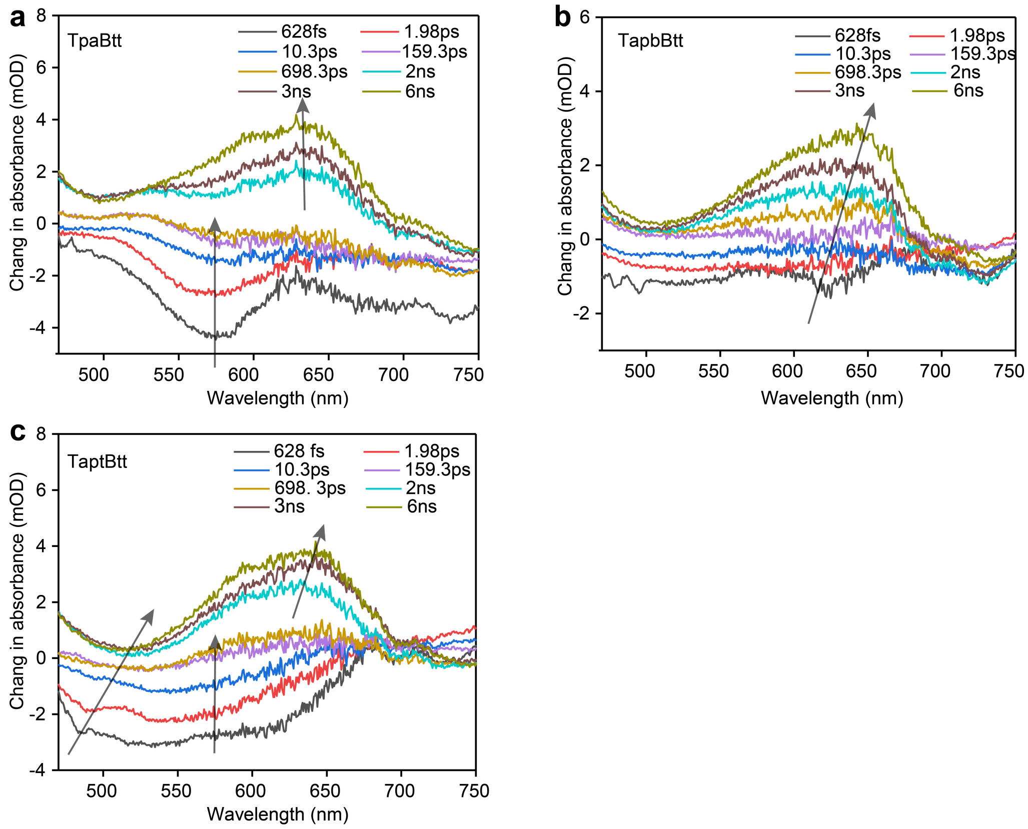

Supplementary Figure 23. Transient absorption spectra. (a) TpaBtt, (b) TapbBtt and (c) TaptBtt.

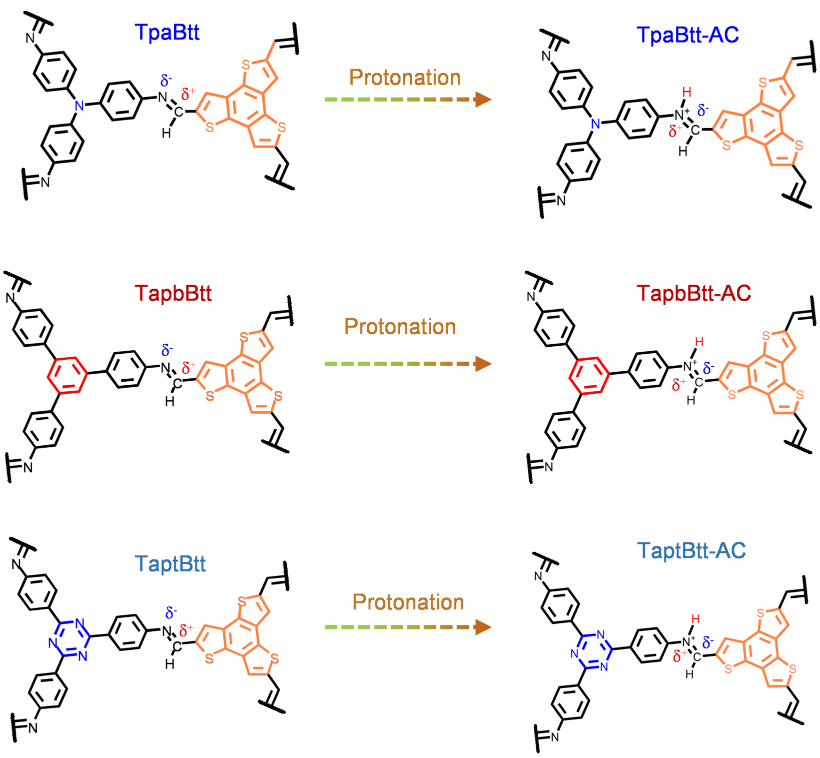

Supplementary Figure 24. Scheme of electron transfer path for both pristine COFs and protonated COFs.

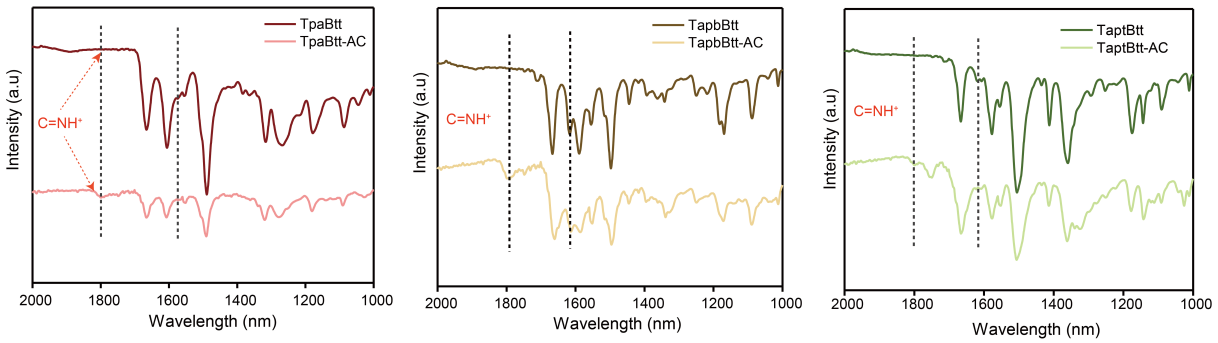

Supplementary Figure 25. Comparison of the FTIR spectra of isomeric pristine COFs and protonated COFs.

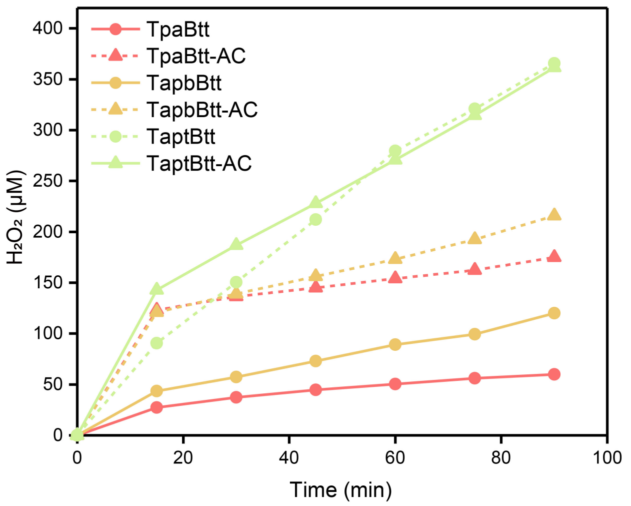

Supplementary Figure 26. Photocatalytic H_2O_2 performance under visible light irradiation for pristine COFs and protonated COFs. Conditions: water (50 mL), catalyst (15 mg), 300 W Xe lamp, λ > 420 nm.

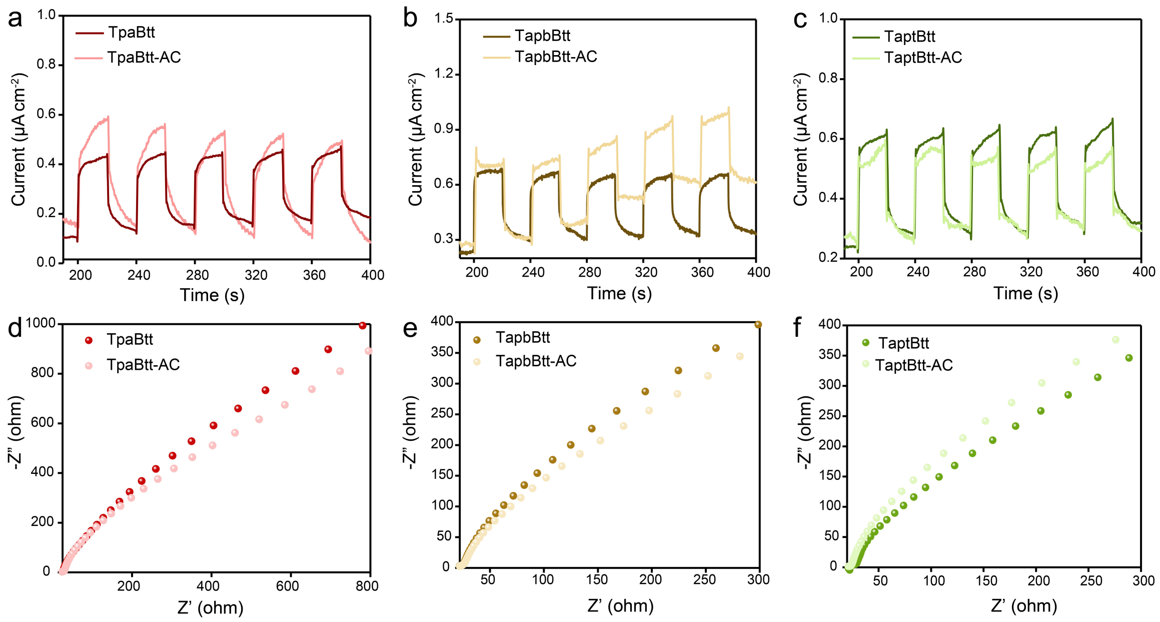

Supplementary Figure 27. Transient photocurrent responses and electrochemical impedance spectroscopy. (a-c) Transient photocurrent responses under visible light irradiation. (d-f) Electrochemical impedance spectroscopy of pristine COFs and protonated COFs.

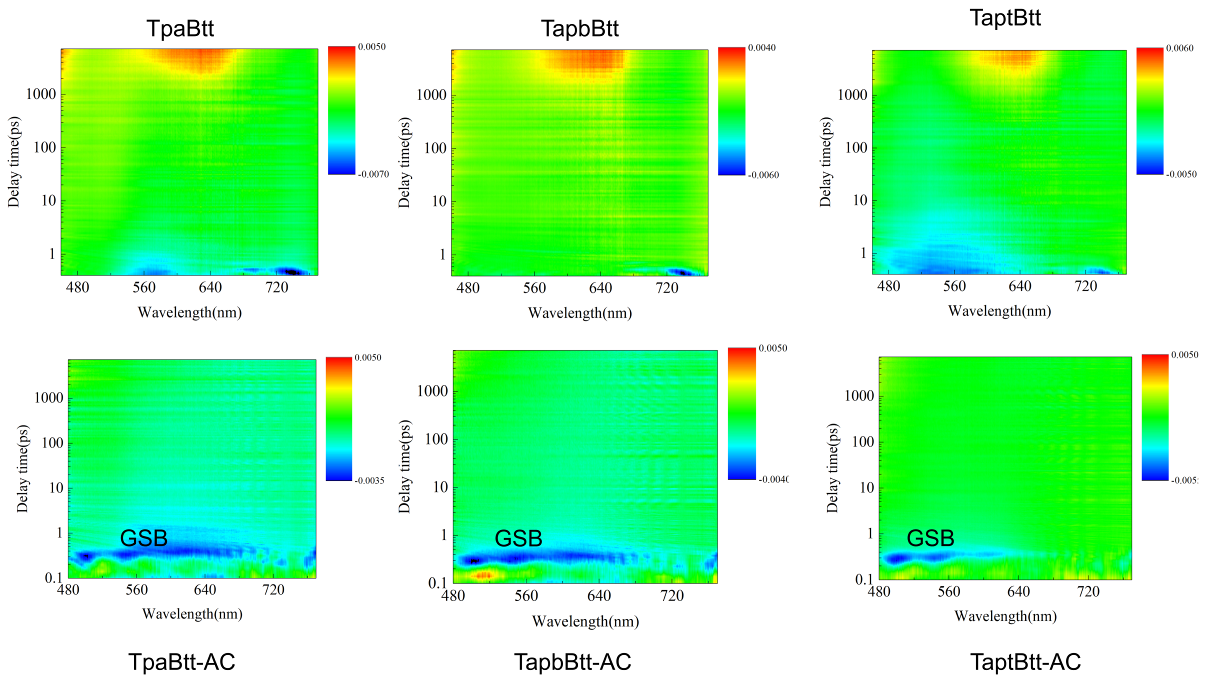

Supplementary Figure 28. 2D contour plots of TA spectra for unprotonated and protonated COFs.

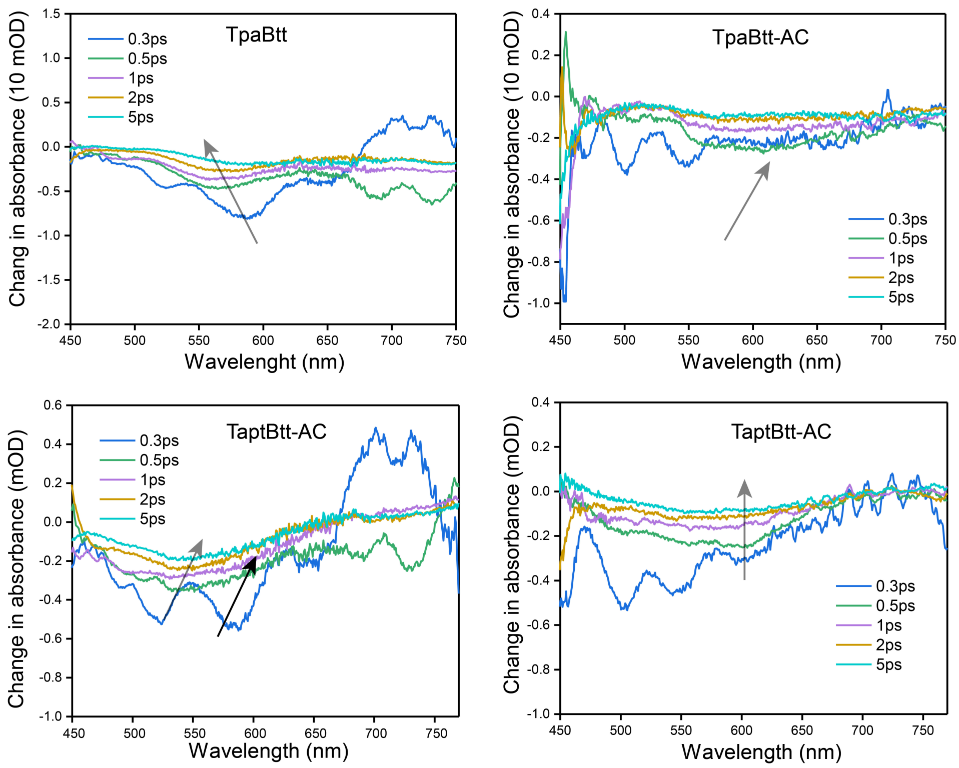

Supplementary Figure 29. Spectral signals of TpaBtt-AC and TaptBtt-AC on the ps timescales compared with pure COFs.

Supplementary Note 4:

As shown in the fs-TA spectra in **Supplementary Figure 28**, after being excited by a pump pulse with a wavelength of 400 nm (consistent with the test conditions of pure COFs), the TpaBtt-AC shows an obvious negative signal at 500-650 compared with pure TpaBtt, which belongs to ground state bleaching. This signal represents the process of donor molecules in the material leaving the ground state to become excited states. The signal of TpaBtt-AC becomes more sustained compared with TpaBtt, indicating that TpaBtt-AC has more excited states formed under photoexcitation. This enables the charge form/transfer in a manner of more ordered way to a certain extent[23]. On the contrary, TaptBtt weakens after protonation, which is consistent with the photoelectrochemical results and the photocatalytic performance. Subsequently, we explored the different spectral features revealed by spectral cross sections at different times and the observed dynamic lines in **Supplementary Figure 29**. TpaBtt-AC showed obvious blue-shift after 0.3 ps around at 550 nm. However, this blue-shift becomes less obvious after protonation, indicating that the short-range push-pull effect is weakened, further demonstrating that the excited electrons of TpaBtt-AC can be efficiently transferred to the receptor unit, rather than being concentrated on the imine bond. For TaptBtt and TaptBtt-AC, the trend of spectral cross section is the opposite of TpaBtt and TpaBtt-AC.

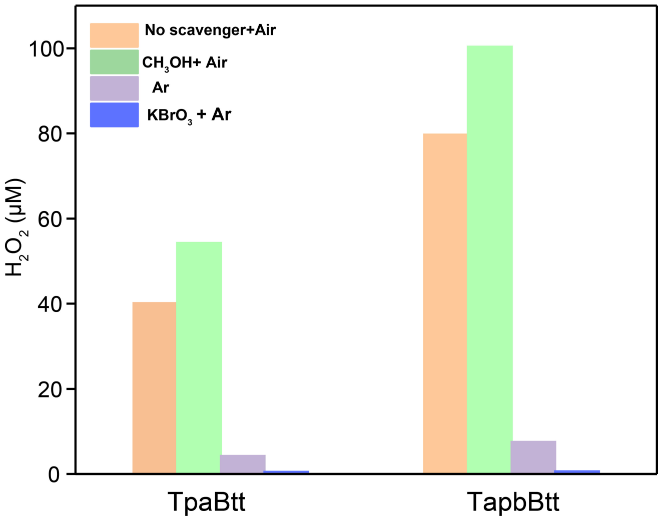

Supplementary Figure 30. Amount of H_2O_2 produced on TpaBtt and TapbBtt in CH_3OH (10% *v*/*v*, as the hole acceptor) with $KBrO_3$ (0.01 M, as the electron acceptor). Conditions: 50 mL H_2O; 15 mg catalyst, 300 W Xe lamp, $\lambda > 420$ nm.

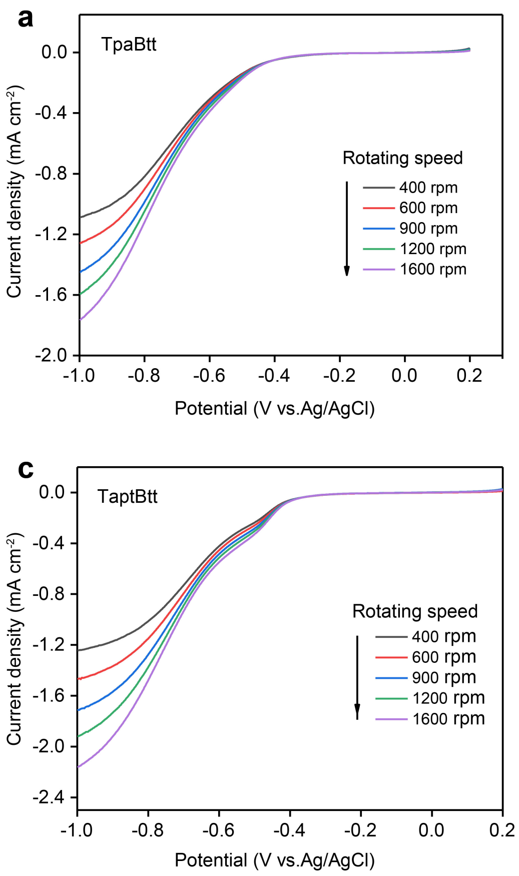

Supplementary Figure 31. Linear-sweep RDE voltammograms. (a) TpaBtt, (b) TapbBtt and (c) TaptBtt measured at different rotating speeds.

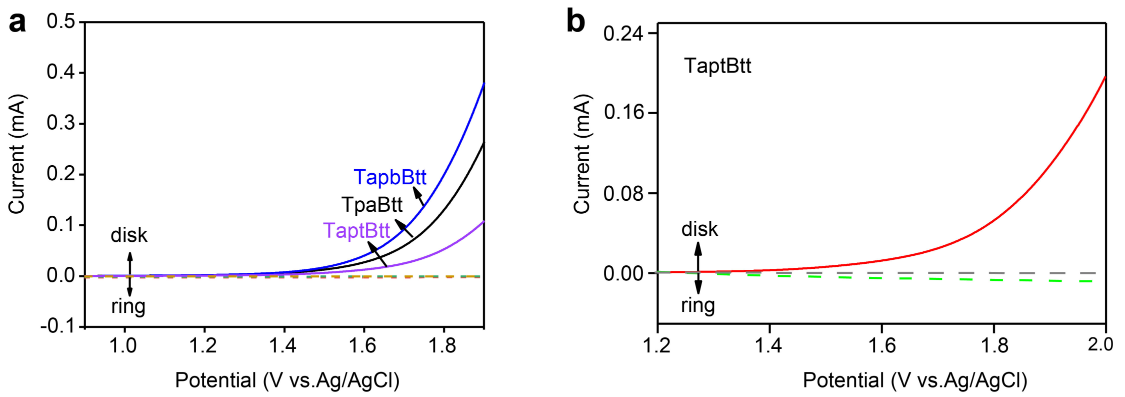

Supplementary Figure 32. RRDE voltammograms. (a) RRDE voltammograms obtained in 0.1 M phosphate buffer solution with a scan rate of 10 mV s^{-1} and a rotation of 1000 rpm. The potential of Pt ring electrode is set at -0.23 V versus Ag/AgCl to detect O_2. (b) RRDE voltammograms obtained in 0.1 M phosphate buffer solution with a scan rate of 10 mV s^{-1} and a rotation rate of 1000 rpm. The potential of Pt ring electrode is set to 0.6 V versus Ag/AgCl to detect H_2O_2. The oxidation current observed during RRDE tests indicates the oxidation of H_2O_2 occurs at the ring electrode.

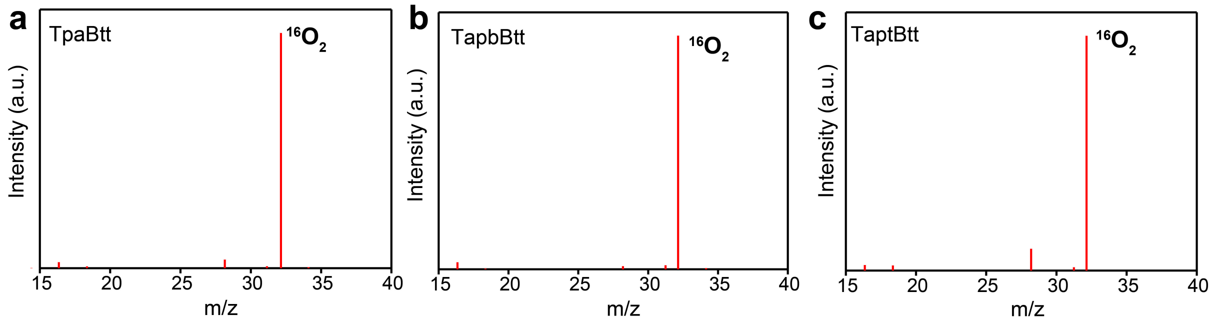

Supplementary Figure 33. $H_2{}^{18}O$ isotope labelling experiments to test whether water is used to produce oxygen under light conditions. (a) TpaBtt, (b) TapbBtt and (c) TaptBtt. The relative intensity of $^{16}O_2$ and $^{18}O_2$ in the headspace of reactive vessels after photoirradiation was measured by GC-MS. The abundance of $^{18}O_2$ is not produced under light conditions, indicating that the three COFs do not use water to produce oxygen via 4 e^- WOR process.

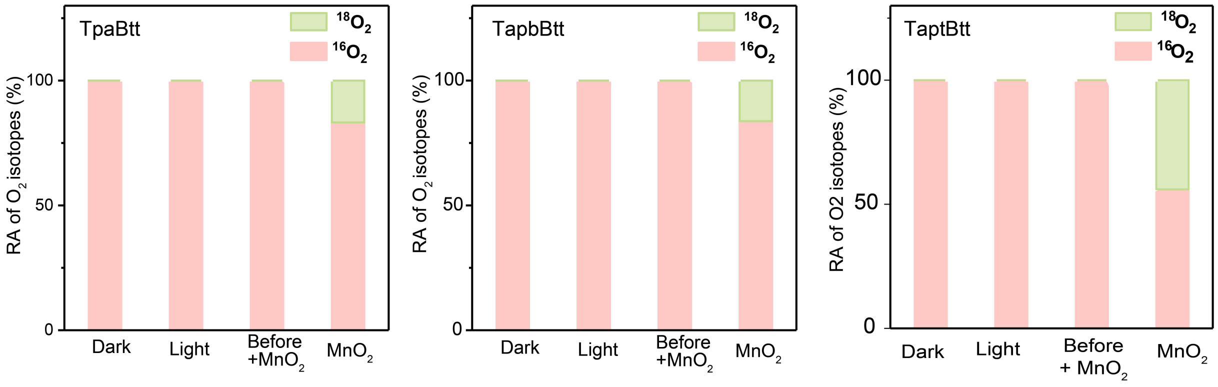

Supplementary Figure 34. Relative intensities of $^{16}O_2$ and $^{18}O_2$ in the gas products decomposed from H_2O_2 generated using different COFs (MnO_2 was used to decompose H_2O_2).

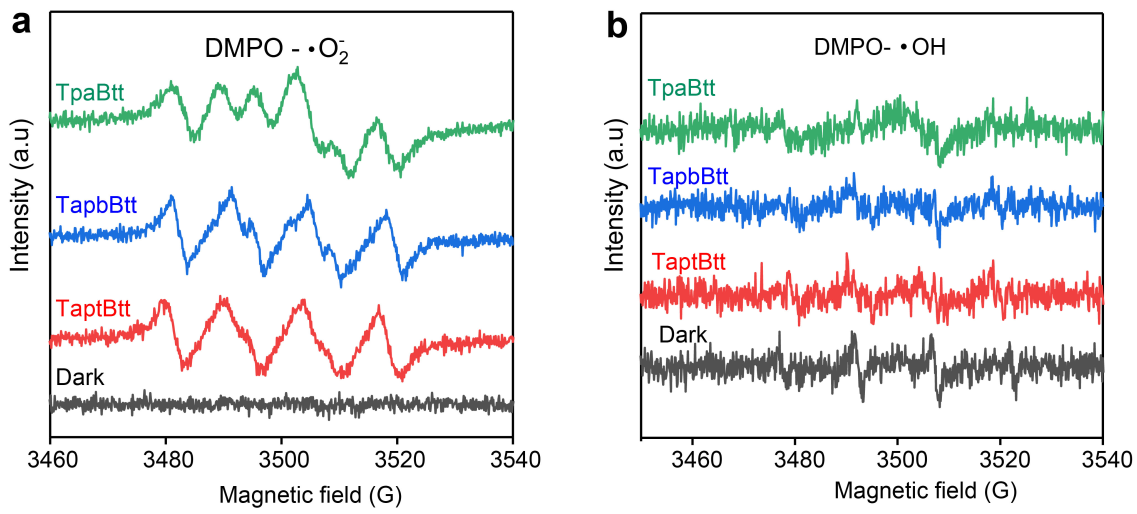

Supplementary Figure 35. DMPO spin trapping EPR spectra. DMPO spin trapping EPR spectra of TpaBtt, TapbBtt and TaptBtt for measuring (a) $\cdot O_2^-$ and (b) ·OH under dark and visible light (10 min). The EPR signals are resulted from DMPO itself rather than photogenerated ·OH by COFs as DMPO-·OH adduct is very easy to form when DMPO is irradiated in the presence of O_2.

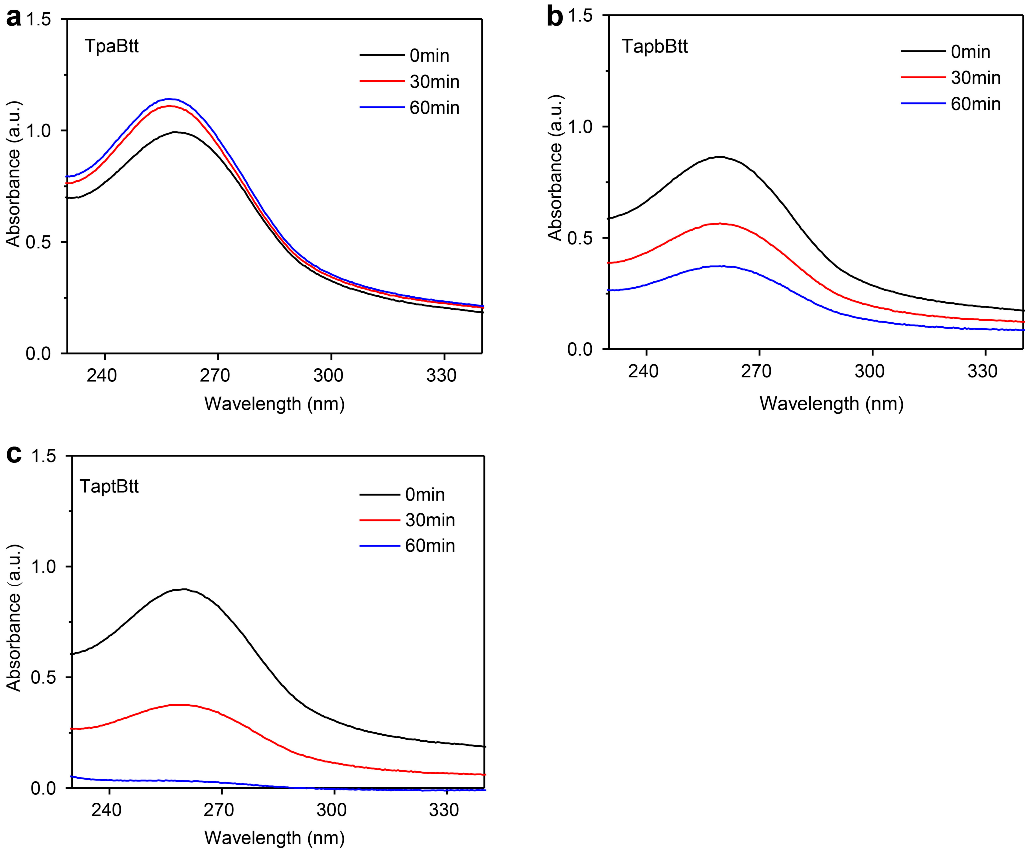

Supplementary Figure 36. Photocatalytic production of $\cdot O_2^-$. Photocatalytic production of $\cdot O_2^-$ on (a) TpaBtt, (b) TapbBtt, and (c) TaptBtt detected by NBT method.

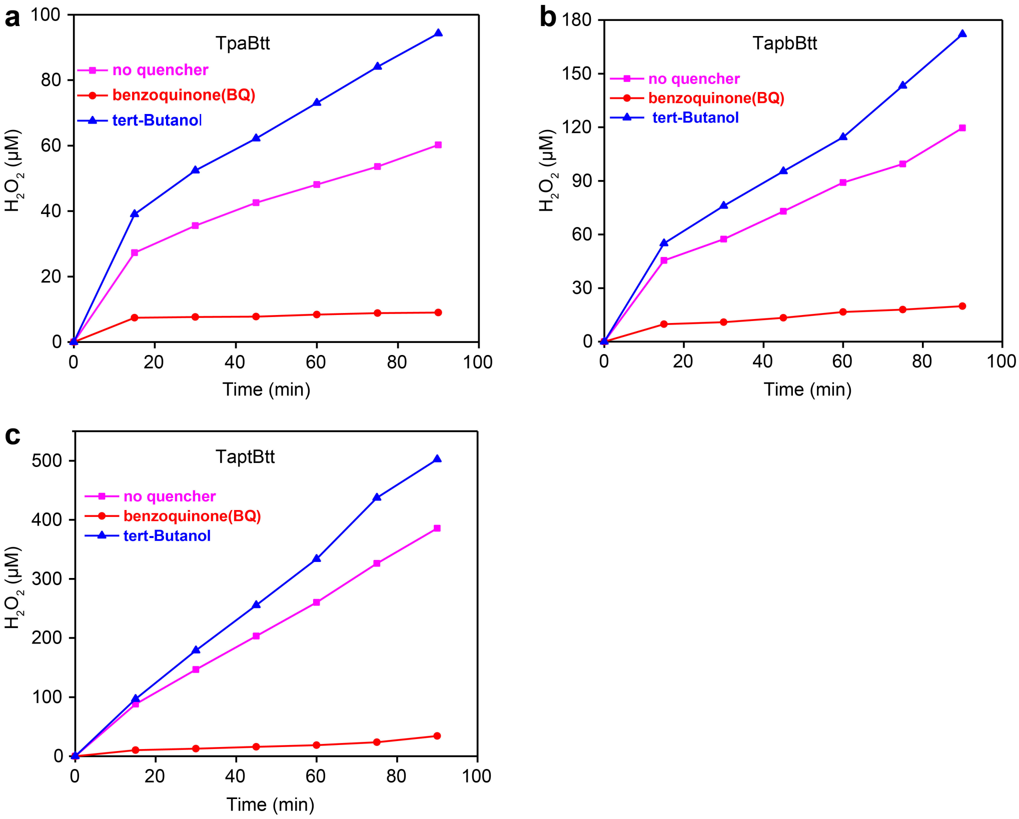

Supplementary Figure 37. Influence of benzoquinone and tert-butanol for the H_2O_2 generation. Influence of benzoquinone (BQ, $\cdot O_2^-$ scavenger) and tert-butanol ($\cdot OH$ scavenger) for the H_2O_2 generation on (a) TpaBtt, (b) TapbBtt and (c) TaptBtt. Conditions: 50 mL H_2O, 15 mg catalyst, 300 W Xe lamp, λ > 420 nm.

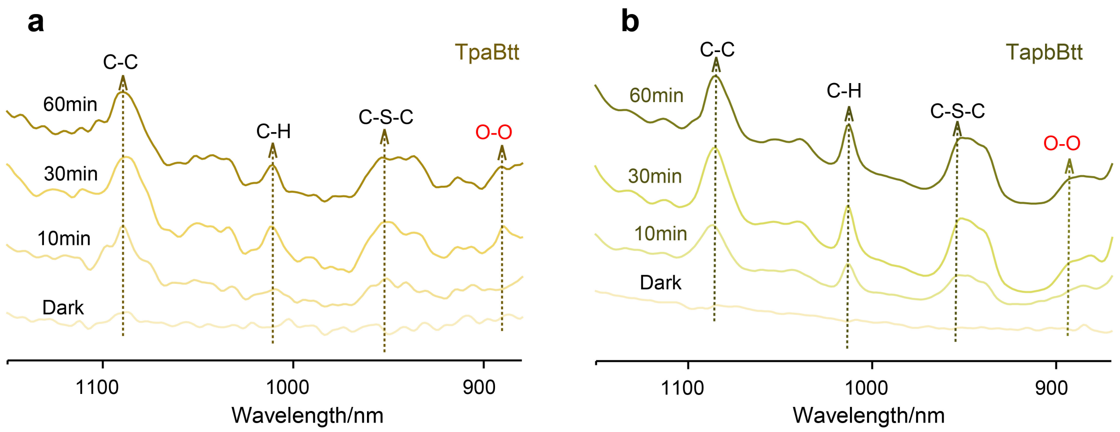

Supplementary Figure 38. *In-situ* DRIFT spectra. *In-situ* DRIFT spectra (range 900-1100 nm) of (a) TpaBtt and (b) TapbBtt.

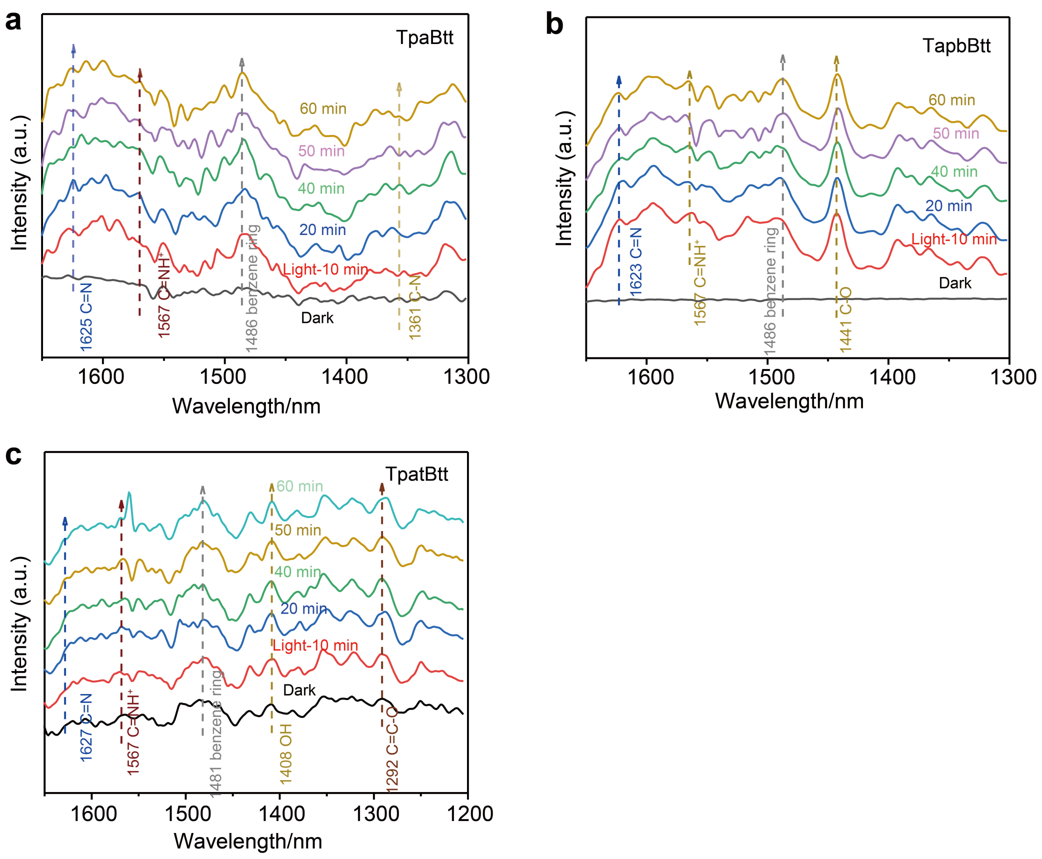

Supplementary Figure 39. *In-situ* DRIFT spectra. *In-situ* DRIFT spectra of (a) TaptBtt, (b) TapbBtt and (c) TpaBtt. The *in-situ* DRIFT measurements were conducted to monitor the active sites for the generation of H_2O_2 in O_2-saturated water under λ > 420 nm irradiation. Vibration corresponding to C=N (1622 cm^{-1}) and benzene ring (1481 cm^{-1}) for three COFs were more and more obvious with the delay of illumination time,[24] indicating that the active site of photocatalytic hydrogen peroxide production is likely to be located on the C atom of the benzene ring in TapbBtt and TaptBtt, and the imine linkage acts as a bridge for electron transfer. Part of structural transformation induced infrared vibration shifted (such as 1356-1360 cm^{-1} for C-N) for TpaBtt.

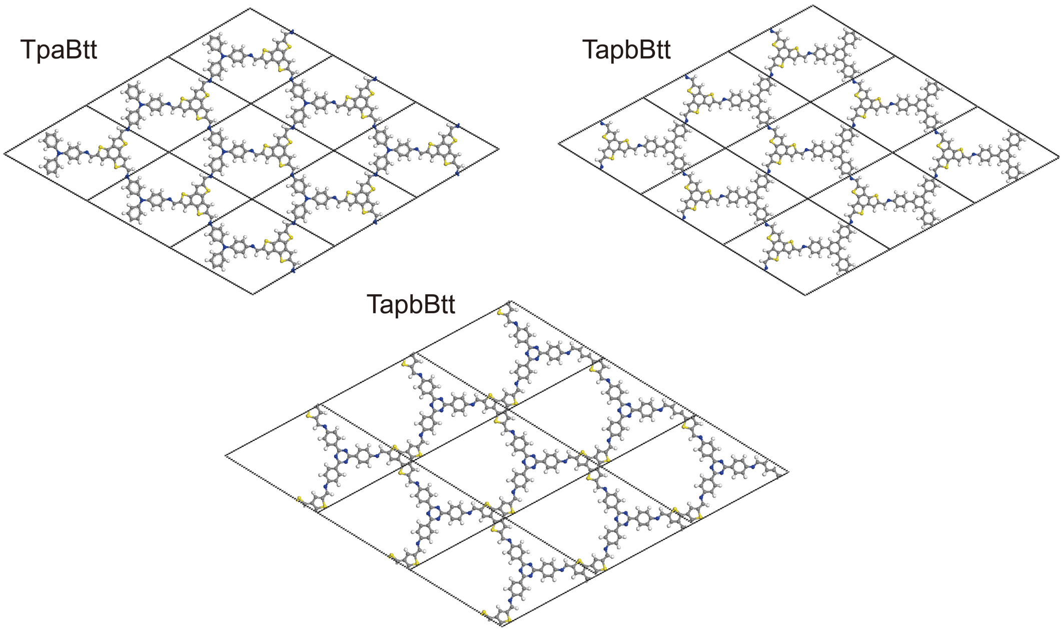

Supplementary Figure 40. Optimal structural models of three COFs.

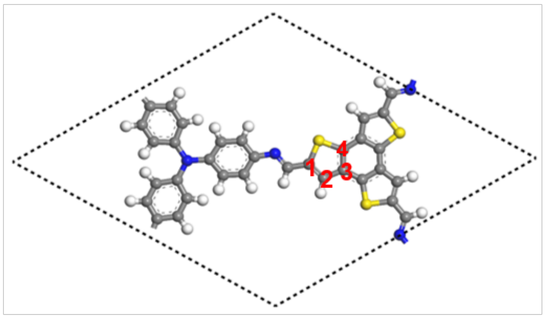

Sitev	C_1	C_2	C_3	C_4	average
TpaBtt	-0.022	-0.059	-0.027	-0.014	-0.122

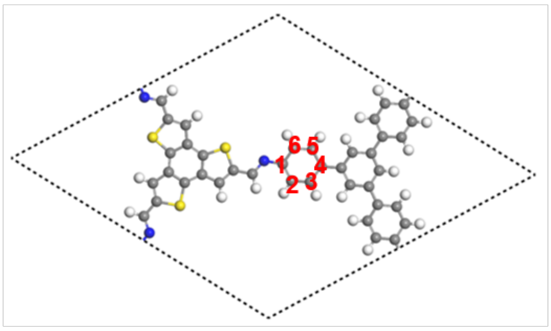

Sitev	C_1	C_2	C_3	C_4	C_5	C_6	average
TapbBtt	0.035	-0.048	-0.041	0.005	-0.042	-0.041	-0.132

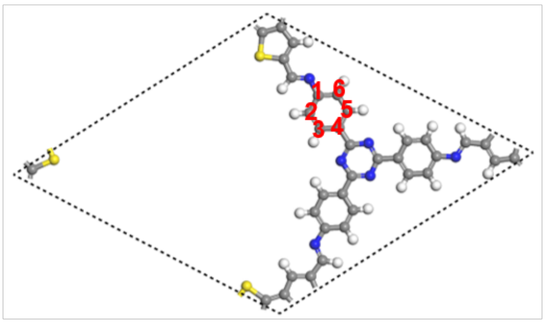

Sitev	C_1	C_2	C_3	C_4	C_5	C_6	average
TaptBtt	0.041	-0.047	-0.033	-0.007	-0.033	-0.04	-0.119

Supplementary Figure 41. Hirshfeld charge of the different carbon atoms at benzene ring for three COFs. The white, grey, blue, yellow and red spheres refer to hydrogen, carbon, nitrogen sulfur and oxygen, respectively.

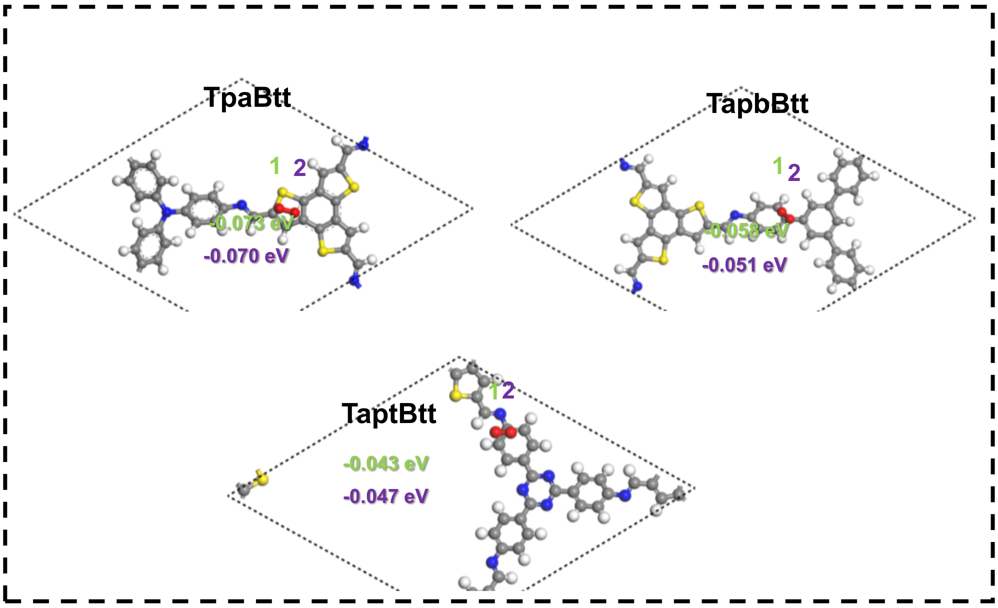

Supplementary Figure 42. Hirshfeld charge of the corresponding O_2 adsorption for three COFs. The white, grey, blue, yellow and red spheres refer to hydrogen, carbon, nitrogen sulfur and oxygen, respectively.

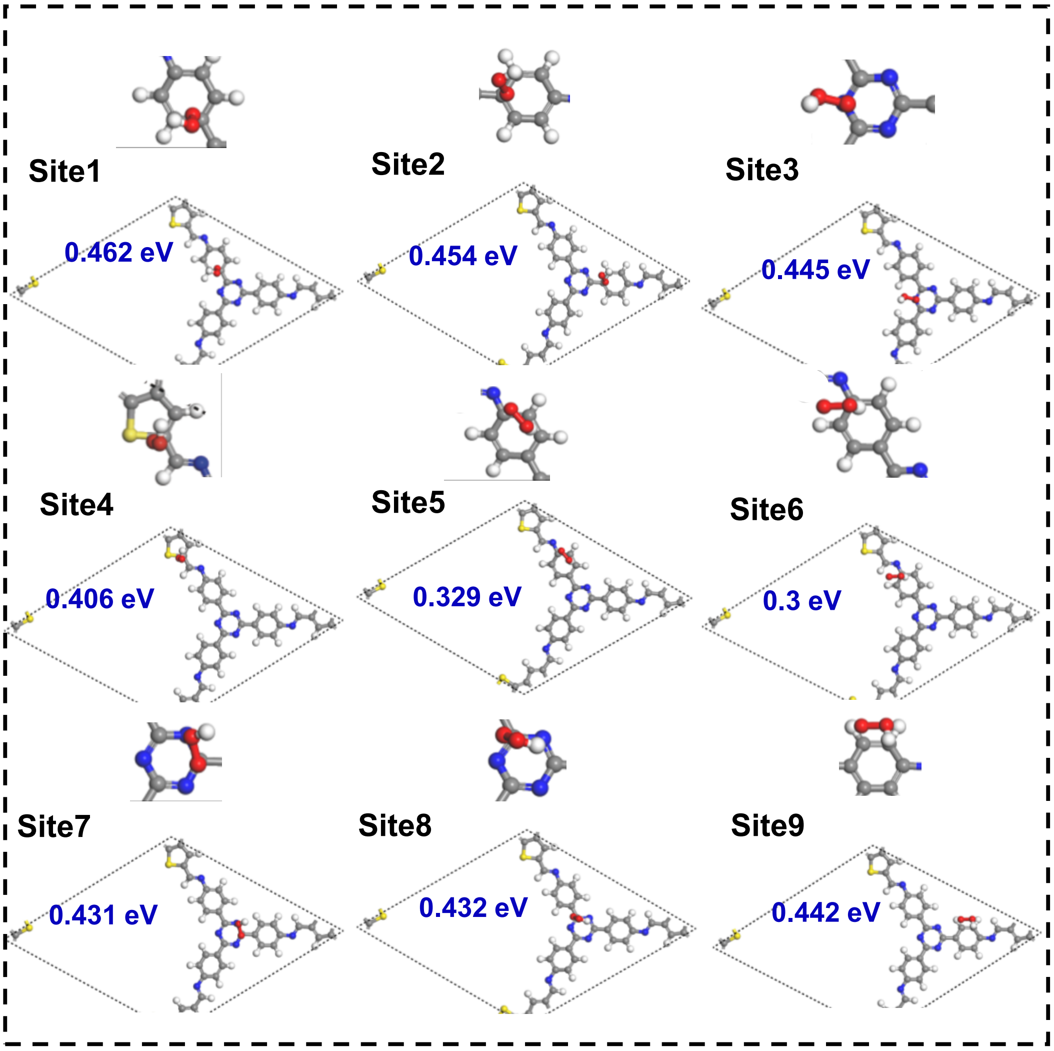

Supplementary Figure 43. Gibbs free energy of OOH* at different active sites for TaptBtt. All structures are optimized for top view. The white, grey, blue, yellow and red spheres refer to hydrogen, carbon, nitrogen sulfur and oxygen, respectively.

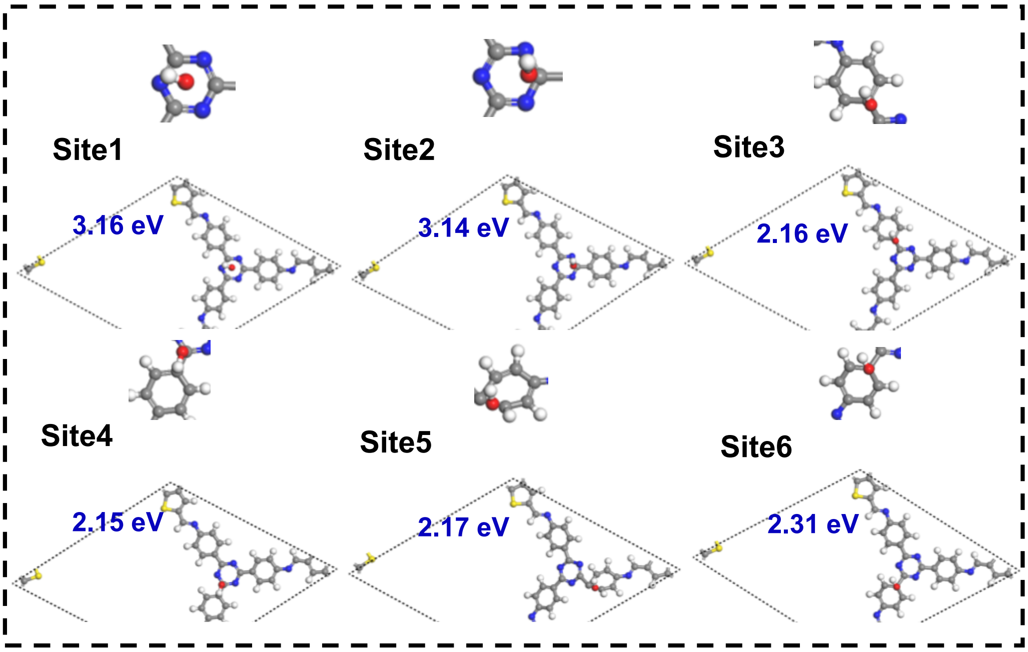

Supplementary Figure 44. Gibbs free energy of *OH at different active sites for TaptBtt. All of structures are optimized for top view. The white, grey, blue, yellow and red spheres refer to hydrogen, carbon, nitrogen sulfur and oxygen, respectively.

Supplementary Table 1. Calculated molecular orbital at different excited states for COFs (Excited states were calculated by the CAM-B3LYP functional and the def2-SVP basis set via ORCA software).

Model	Excitation	MOT	MO contribution of transition (%)	E(eV) [λ] (nm)	f
TpaBtt	S0 → S1	HOMO-4 → LUMO	1.19	378 [3.28]	1.2955
		HOMO-3 → LUMO	3.35		
		HOMO-1 → LUMO	6.12		
		HOMO → LUMO	79.72		
	S0 → S2	HOMO-2 → LUMO	68.25	310 [4.00]	0.0151
		HOMO-2 → LUMO+2	5.59		
		HOMO-1 → LUMO+1	13.25		
		HOMO → LUMO+1	4.68		
	S0 → S3	HOMO-9 → LUMO	13.80	304 [4.08]	0.1496
		HOMO-4 → LUMO	8.70		
		HOMO-3 → LUMO	5.82		
		HOMO-2 → LUMO	13.09		
		HOMO-2 → LUMO+1	2.75		
		HOMO-1 → LUMO	54.81		
		HOMO → LUMO+2	10.06		
	S0 → S4	HOMO-4 → LUMO	1.29	290 [4.27]	0.0274
		HOMO-3 → LUMO	1.08		
		HOMO-3 → LUMO+6	1.04		
		HOMO-1 → LUMO	5.56		
		HOMO-1 → LUMO+3	3.44		
		HOMO → LUMO	1.49		
		HOMO → LUMO+3	65.84		
		HOMO → LUMO+5	1.98		
		HOMO → LUMO+6	1.64		

Model	Excitation	MOT	MO contribution of transition (%)	E(eV) [λ] (nm)	f
TapbBtt	S0 → S1	HOMO-9 → LUMO	4.00	354 [3.51]	1.3681
		HOMO-4 → LUMO	3.51		
		HOMO → LUMO	82.86		
	S0 → S2	HOMO-2 → LUMO+2	4.34	311 [3.99]	0.01853
		HOMO-1 → LUMO	71.04		
		HOMO → LUMO+2	11.41		
	S0 → S3	HOMO-10 → LUMO	9.26	304 [4.08]	0.1234
		HOMO-9 → LUMO	12.91		
		HOMO-5 → LUMO	6.26		
		HOMO-4 → LUMO	5.80		
		HOMO-2 → LUMO	41.86		
		HOMO → LUMO	7.05		
	S0 → S4	HOMO-9 → LUMO+1	3.33	271 [4.58]	0.0013
		HOMO-3 → LUMO	16.49		
		HOMO-3 → LUMO+3	24.96		
		HOMO-2 → LUMO+1	19.61		
		HOMO → LUMO+1	17.21		

Model	Excitation	MOT	MO contribution of transition (%)	E(eV) [λ] (nm)	f
TaptBtt	S0 → S1	HOMO-2 → LUMO	12.13	356 [3.49]	1.4997
		HOMO → LUMO	73.35		
	S0 → S2	HOMO-1 → LUMO	63.38	314 [3.94]	0.0209
		HOMO-1 → LUMO+2	15.01		
		HOMO → LUMO+3	11.61		
	S0 → S3	HOMO-13 → LUMO	4.68	306 [4.05]	0.0829
		HOMO-11 → LUMO	12.62		
		HOMO-2 → LUMO	40.19		
		HOMO-2 → LUMO+2	6.38		
		HOMO → LUMO	11.45		
	S0 → S4	HOMO-10 → LUMO+1	28.26	278 [4.45]	0.0007
		HOMO-9 → LUMO	31.99		
		HOMO-9 → LUMO+2	30.34		

Supplementary Table 2. Atomistic coordinates of AA-stacking mode of the simulated TpaBtt, TapbBtt and TaptBtt. All cif files of COFs have been stored in the CoRE-COF-Database.

TpaBtt

Space group: P-6

a=b=19.1881 Å, c=3.5238 Å

α=β=90°, γ=120°

Atom	x/a	y/b	z/c
C	0.24461	0.61648	0.50000
C	0.19800	0.53386	0.50000
C	0.35492	0.54745	0.50000
C	0.50903	0.62754	0.50000
C	0.47460	0.55031	0.50000
C	0.39666	0.50974	0.50000
N	0.48367	0.00474	0.50000
C	0.55536	0.05447	0.50000
S	0.65798	0.48708	0.50000
C	0.55594	0.42046	0.50000
C	0.53492	0.34711	0.50000
C	0.59913	0.33509	0.50000
C	0.66795	0.40303	0.50000
H	0.20700	0.48863	0.50000
H	0.30203	0.50375	0.50000
H	0.57115	0.66030	0.50000
H	0.36662	0.44836	0.50000
H	0.59827	0.03767	0.50000
H	0.47632	0.30135	0.50000
N	0.33333	0.66667	0.50000

TapbBtt

Space group: P-6

a=b=21.3500 Å, c=3.5050 Å

α=β=90°, γ=120°

Atom	x/a	y/b	z/c
C	0.34139	0.73543	0.00000
C	0.27216	0.67538	0.00000
S	0.46178	0.63876	0.00000
C	0.39685	0.54778	0.00000
C	0.32674	0.53218	0.00000
C	0.42567	0.50220	0.00000
N	0.49557	0.53505	0.00000
C	0.52810	0.49395	0.00000
C	0.60336	0.53107	0.00000
C	0.48780	0.41843	0.00000
C	0.52335	0.38083	0.00000
C	0.59864	0.41667	0.00000
C	0.63876	0.49233	0.00000
C	0.70717	0.40752	0.00000
C	0.63300	0.37401	0.00000
H	0.28002	0.47773	0.00000
H	0.38890	0.44346	0.00000
H	0.63181	0.59001	0.00000
H	0.42906	0.38895	0.00000
H	0.49213	0.32221	0.00000
H	0.69752	0.52155	0.00000
H	0.73819	0.46586	0.00000

TapTBtt

Space group: P-6

a=b=21.7550 Å, c=3.4930 Å

α=β=90°, γ=120°

Atom	x/a	y/b	z/c
C	0.04763	0.97473	0.50000
C	0.07247	0.04697	0.50000
S	0.11231	0.95216	0.50000
C	0.17305	0.04133	0.50000
C	0.14455	0.08564	0.50000
C	0.24930	0.06830	0.50000
C	0.59481	0.29474	0.50000
N	0.63357	0.26185	0.50000
C	0.51668	0.25281	0.50000
C	0.47641	0.28700	0.50000
C	0.40260	0.24729	0.50000
C	0.36780	0.17332	0.50000
C	0.40765	0.13865	0.50000
C	0.48160	0.17824	0.50000
N	0.29209	0.13638	0.50000
H	0.17512	0.14305	0.50000
H	0.26783	0.03063	0.50000
H	0.50203	0.34438	0.50000
H	0.37178	0.27386	0.50000
H	0.38263	0.08157	0.50000
H	0.51146	0.15062	0.50000

Supplementary Table 3. N_2 adsorption-desorption analysis of three COFs.

Catalysts	BET surface area (m^2 g^{-1})	Total pore volume (cm^3 g^{-1})	Micropore volume (cm^3 g^{-1})	Pore size (nm)
**TpaBtt**	850.88	0.42	0.28	1.20
**TapbBtt**	1493.38	0.71	0.55	1.45
**TaptBtt**	994.95	0.53	0.36	1.51

Supplementary Table 4. Comparison of photocatalytic H_2O_2 production by reported photocatalysts (O_2-saturated pure water: continuous bubbled into O_2 before illumination; continuous aeration: continuous bubbled into O_2 during illumination; pure water: not bubbled into extra O_2).

Photocatalysts	Conditions	H_2O_2 production rates (μmol h^{-1})	Reaction pathways	Ref
g-C_3N_4 /PDI_{51}	O_2-saturated pure water, 1.67 g/L, 26.9 mW cm^{-2}	1.05	Two-electron O_2 reduction & four-electron water oxidization	25
CN aerogels	O_2-saturated pure water, 1.67 g/L, λ > 420 nm	1.44	/	26
OCN-500	O_2-saturated pure water, 1 g/L, 35.2 mW cm^{-2}	5.3	Two-electron O_2 reduction & four-electron water oxidization	6
g-C_3N_4 (Nv-C≡N-CN)	Continuous aeration, 1 g/L, AM 1.5G, 100 mW cm^{-2}	6.46	One electron O_2 reduction & four-electron water oxidization	27
COF-TpBpy	Pure water, 1.5 g/L, λ > 420 nm	10.4	Two-electron O_2 reduction & two-electron water oxidization	24
CTF-EDDBN	O_2-saturated pure water, 0.6 g/L, 44.5 mW cm^{-2}	2.9	Two-electron O_2 reduction & two-electron water oxidization	11
CTF-BDDBN	O_2-saturated pure water, 2 g/L, AM 1.5G,100 mW cm^{-2}	26.6	Two-electron O_2 reduction & two-electron water oxidization	

PEI/ C_3N_4	Continuous aeration, 1 g/L, AM 1.5G, 100 mW cm^{-2}	4.16	One electron O_2 reduction	[28]
COF-TTA-DHTA	Pure water, 0.3 g/L, Blue LED λ > 420 nm	15.3	-	[29]
sonoCOF-F2	Pure water, 0.6 g/L, Oriel Solar Simulator, 1.0 sun	5.6	-	[30]
Ti-COF	O_2-saturated pure water, 0.3 g/L λ > 420 nm	9.4	-	[31]
TpaBtt	Pure water 1.2 g/L, AM 1.5G 100 mW cm^{-2}	7.8	One electron O_2 reduction	This work
TapbBtt	Pure water, 1.2g/L, AM 1.5G 100 mW cm^{-2}	9.9		This work
TaptBtt	Pure water, 1.2 g/L, AM 1.5G 100 mW cm^{-2}	28.6	One-electron O_2 reduction & 2e-WOR	This work

Supplementary references

1. Taerum, T., Lukoyanova, O. Wylie, R. G. & Perepichka, D. F. Synthesis, polymerization, and unusual properties of new star-shaped thiophene oligomers. *Org. Lett.* **11**, 3230-3233 (2009).
2. Li, Z. *et al.* Three-component donor–π–acceptor covalent–organic frameworks for boosting photocatalytic hydrogen evolution. *J. Am. Chem. Soc.* **145**, 8364-8374 (2023).
3. Wang, H. *et al.* Integrating suitable linkage of covalent organic frameworks into covalently bridged inorganic/organic hybrids toward efficient photocatalysis. *J. Am. Chem. Soc.* **142**, 4862-4871 (2020).
4. Zhang, F.-M. *et al.* Rational design of MOF/COF hybrid materials for photocatalytic H_2 evolution in the presence of sacrificial electron donors. *Angew. Chem. Int. Ed.* **57**, 12106-12110 (2018).
5. Teng, Z. *et al.* Atomically dispersed antimony on carbon nitride for the artificial photosynthesis of hydrogen peroxide. *Nat. Cat.* **4**, 374-384 (2021).
6. Wei, Z. *et al.* Efficient visible-light-driven selective oxygen reduction to hydrogen peroxide by oxygen-enriched graphitic carbon nitride polymers. *Energy Environ. Sci.* **11**, 2581-2589 (2018).
7. Shiraishi, Y. *et al.* Resorcinol–formaldehyde resins as metal-free semiconductor photocatalysts for solar-to-hydrogen peroxide energy conversion. *Nat. Mater.* **18**, 985-993 (2019).
8. Lazaridis, T. *et al.* Capabilities and limitations of rotating disk electrodes versus membrane electrode assemblies in the investigation of electrocatalysts. *Nat. Catal.* **5**, 363-373 (2022).
9. Cheng, H. *et. al*. Rational design of covalent heptazine frameworks with spatially separated redox centers for high-efficiency photocatalytic hydrogen peroxide production. *Adv. Mater.* **34**, 2107480 (2022).
10. Jung, E. *et al.* Atomic-level tuning of Co–N–C catalyst for high-performance electrochemical H_2O_2 production. *Nat. Mater.* **19**, 436-442 (2020).

11. Chen, L. *et al.* Acetylene and diacetylene functionalized covalent triazine frameworks as metal-free photocatalysts for hydrogen peroxide production: A new two-electron water oxidation pathway. *Adv. Mater.* **32**, 1904433 (2020).

12. Hülsey, M. J. *et al.* In situ spectroscopy-guided engineering of rhodium single-atom catalysts for CO oxidation. *Nat. Commun.* **10**, 1330 (2019).

13. Jiang, H. Y. *et al.* Copper-based coordination polymer nanostructure for visible light photocatalysis. *Adv. Mater.* **28**, 9776-9781 (2016).

14. Weigend, F. & Ahlrichs, R. Balanced basis sets of split valence, triple zeta valence and quadruple zeta valence quality for H to Rn: Design and assessment of accuracy. *Phys. Chem. Chem. Phys.* **7**, 3297-3305 (2005).

15. Weigend, F. Accurate Coulomb-fitting basis sets for H to Rn. *Phys. Chem. Chem. Phys.* **8**, 1057-1065 (2006).

16. Calais, J.-L. Density-functional theory of atoms and molecules. *Inter. J. Quantum Chem.* **47**, 101-101 (1993).

17. Grimme, S. *et al*. Effect of the damping function in dispersion corrected density functional theory. *J. Comp. Chem.* **32**, 1456-1465 (2011).

18. Yanai, T. *et al*. A new hybrid exchange–correlation functional using the Coulomb-attenuating method (CAM-B3LYP). *Chem. Phys. Lett.* **393**, 51-57 (2004).

19. Dong, W. *et al.* Isomeric oligo(phenylenevinylene)-based covalent organic frameworks with different orientation of imine bonds and distinct photocatalytic activities. *Angew. Chem. Int. Ed.* **62**, e202216073 (2023).

20. Patterson, A. L. The Scherrer formula for X-ray particle size determination. *Phys. Rev.* **56**, 978-982 (1939).

21. Kochergin, Y. S. *et al*. Multifunctional visible-light powered micromotors based on semiconducting sulfur- and nitrogen-containing donor-acceptor polymer. *Adv. Funct. Mater.* **30**, 2002701 (2020).

22. Li, W. *et al.* A "chain-lock" strategy to construct a conjugated copolymer network for supercapacitor applications. *J. Mater. Chem. A* **7**, 116-123 (2019).

23. Cheng, C. *et al*. Verifying the charge-transfer mechanism in S-scheme heterojunctions using femtosecond transient absorption spectroscopy. *Angew. Chem. Int. Ed.* **62**, e202218688 (2023).
24. Kou, M. *et al.* Molecularly engineered covalent organic frameworks for hydrogen peroxide photosynthesis. *Angew. Chem. Int. Ed.* **61**, e202200413 (2022).
25. Shiraishi, Y. *et al.* Sunlight-driven hydrogen peroxide production from water and molecular oxygen by metal-free photocatalysts. *Angew. Chem. Int. Ed.* **53**, 13454-13459 (2014).
26. Ou, H. *et al*. Carbon nitride aerogels for the photoredox conversion of water. *Angew. Chem. Int. Ed.* **56**, 10905-10910 (2017).
27. Zhang, X. *et al.* Unraveling the dual defect sites in graphite carbon nitride for ultra-high photocatalytic H_2O_2 evolution. *Energy. Environ. Sci.* **15**, 830-842 (2022).
28. Zeng, X. *et al.* Simultaneously tuning charge separation and oxygen reduction pathway on graphitic carbon nitride by polyethylenimine for boosted photocatalytic hydrogen peroxide production. *ACS Catal.* **10**, 3697-3706 (2020).
29. Tan, F. *et al.* Aqueous synthesis of covalent organic frameworks as photocatalysts for hydrogen peroxide production. *CCS Chem.* **4**, 3751-3741 (2021).
30. Zhao, W. *et al.* Accelerated synthesis and discovery of covalent organic framework photocatalysts for hydrogen peroxide production. *J. Am. Chem. Soc.* **144**, 9902-9909 (2022).
31. Han, W.-K. *et al.* Targeted construction of a three-dimensional metal covalent organic framework with spn topology for photocatalytic hydrogen peroxide production. *Chem. Eng. J.* **449**, 137802 (2022).
